# Acute requirement for the hippocampus in putatively conscious vision revealed by a mouse model of blindsight

**DOI:** 10.1016/j.cub.2026.03.031

**Published:** 2026-04-09

**Authors:** Nikhil Bhatla, Kathryn Cheong, Sho Takahashi, Corliss Lay, Matthew Fiedeldey, Kathleen Liang, Mo Mao, Yun-Fan Wu, Sandy Lu, Karis Yun, Viveca Ganti, Raymond Tsao, Xiaozhou Zhang, Kenzy A. Mohamed, Yul Chang, Yucong Geng, Casey Dai, Ashley K. Liu, Yee May Lwin, Heidi Kim, Vamsi Subraveti, Narumi Mitchell, Na Youn Jo, Umais Khan, Roger Anguera-Singla, Zephyr Weinreich, Hanna E. Willis, Holly Bridge, Michael P. Stryker, Hillel Adesnik

**Affiliations:** 1 Institute for All Minds, Berkeley, CA 94704, USA; 2 Department of Molecular and Cellular Biology, University of California, Berkeley, Berkeley, CA 94720, USA; 3 Helen Wills Neuroscience Institute, University of California, Berkeley, Berkeley, CA 94720, USA; 4 Miller Institute for Basic Research, University of California, Berkeley, Berkeley, CA 94720, USA; 5 Department of Physiology, University of California, San Francisco, San Francisco, CA 94158, USA; 6 Department of Neurology, Neuroscape and Weill Institute for Neurosciences, University of California, San Francisco, San Francisco, CA 94158, USA; 7 Swarthmore College, Swarthmore, PA 19081, USA; 8 Centre for Integrative Neuroimaging, Centre for Functional Magnetic Resonance Imaging of the Brain, Nuffield Department of Clinical Neuroscience, University of Oxford, Oxford OX3 9DU, UK; 9 Lead contact

## Abstract

The phenomenon of blindsight provides a unique opportunity to uncover brain areas important for conscious vision. Patients with blindsight lose the conscious experience of seeing while still being able to detect and locate visual stimuli. Blindsight occurs after damage to the primary visual cortex (V1). Rodents are likewise able to detect and locate visual stimuli after damage to V1, though whether they lose conscious vision as humans do is unclear. Here, we report the first mouse model of blindsight that provides evidence that removal of V1 causes mice to lose their putatively conscious vision. This loss occurs only if dLGN, a brain area in the thalamus that connects to V1, is damaged in addition to V1. We use this model to discover that suppressing the hippocampus acutely causes blindsight-like behavior. This suggests that the hippocampus functions in the intact brain to support conscious, but not unconscious, vision. Furthermore, while single, selective ablations of V1 or the hippocampus have only a minor effect on vision, simultaneous ablation causes blindsight-like behavior. These results suggest that V1 and the hippocampus can compensate for the permanent loss of the other for conscious vision, revealing a novel form of plasticity that supports conscious vision. Although never causally implicated in conscious visual perception, the hippocampus emerges as a candidate brain area critical for conscious experience. More broadly, this mouse model of blindsight will enable the interrogation and identification of the neural circuits that underlie conscious and unconscious vision.

## In brief

How does the brain cause conscious experience? Bhatla et al. develop and leverage a mouse model of blindsight to uncover a critical role for the hippocampus in the putatively conscious vision of the mouse. They also reveal compensatory functions for the hippocampus, V1, and dLGN in support of conscious vision.

## Graphical abstract

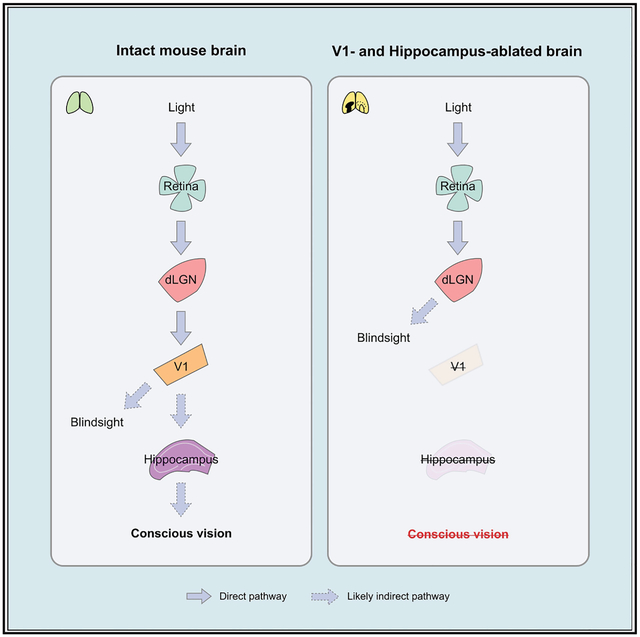

## INTRODUCTION

Each person can know the state of their body and their environment both as a set of facts (e.g., it is 75°F outside) and as a set of consciously felt experiences (e.g., it feels warm outside). Despite the pervasive nature of these conscious experiences, which are referred to as “qualia,” the physical processes that cause them remain mysterious. Empirical and theoretical work has connected a variety of brain areas and activity patterns to subjective experience,^1–8^ but testing whether these cause conscious experiences in humans remains challenging because the causal interventions that would be required are imprecise, of insufficient scale, or unjustifiably risky to a person’s health. Classical paradigms that use special stimuli to manipulate conscious perception in humans, such as near-threshold stimuli,^9^ masking,^10^ and binocular rivalry,^11^ have also been demonstrated in animals,^12–15^ paving the way for causal testing of the cellular correlates of an animal’s putatively conscious vision.

To most efficiently isolate the properties of neural circuits that cause conscious perception, the ideal paradigm would hold behavior constant while conscious experience varied and then would compare the neural circuits required for the conscious perception of a sensory stimulus with those required for the unconscious perception of the same stimulus.^16^ The phenomenon of blindsight is uniquely suited for studying conscious perception because it demonstrates a clear contrast at the neural level between conscious and unconscious vision^16,17^ (but see Phillips^18^). After unilateral damage to the primary visual cortex (V1), patients report that they are blind in the contralateral visual field, a condition known as a scotoma or hemianopia.^19,20^ Importantly, many patients can nonetheless respond appropriately to certain visual stimuli in their blind field, thereby exhibiting blindsight.^21–26^ The phenomenon of blindsight suggests that conscious vision requires V1 but that blindsight (unconscious vision) relies on other brain areas, such as the superior colliculus (SC), the lateral geniculate nucleus (LGN) of the thalamus, and higher visual areas in the cortex.^25,27–29^ Approximately 60% of V1-damaged patients who lack conscious vision have blindsight.^25,26,30^

Since the discovery of blindsight over 50 years ago, researchers have yet to identify brain areas outside of V1 that are required specifically for conscious vision. While several studies have identified neural activity that correlates with conscious vision in brain areas outside of V1 in humans,^31–36^ monkeys,^14,37–42^ and birds,^15^ including in the frontal cortex, there is no evidence that the lesion or inactivation of these regions substantially impairs conscious vision.^43–45^

Although not often associated with visual perception, the hippocampus is a multisensory structure that an animal depends on to locate itself in space using visual cues.^46,47^ Patients with bilateral damage to the hippocampus are impaired in forming new declarative memories,^48^ which are accessible to conscious recollection, and in visually imagining new experiences^49,50^ (but see Squire et al.^51^). Neuroanatomical tracing in the primate brain places the hippocampus at the top of the visual cortical hierarchy,^52^ and hippocampal neurons can be remarkably selective for specific visual objects^53^ and their conscious recognition.^54^ For these reasons, the hippocampus might play a critical role in conscious vision. Francis Crick entertained this hypothesis but ultimately rejected it because patients with hippocampal damage report no loss of conscious vision.^55^ However, it is well known that during the days and weeks following injury (the chronic phase), alternate areas can functionally compensate for those that are lost.^56–58^ By contrast, immediately following injury (the acute phase), effects on perception and behavior can be much more severe, likely because compensatory mechanisms have not yet intervened. Therefore, we hypothesized that the hippocampus plays a role in conscious vision in the intact brain, but that after prolonged injury, another brain area compensates for its loss, masking its normally critical role. To test this hypothesis, we developed an animal model of blindsight.

The principal challenge in developing an animal model of consciousness is the difficulty of measuring an animal’s subjective experience. For humans, a verbal report is taken as ground truth evidence of conscious vision, but animals need an alternative way to report. Several studies have overcome this challenge to test for blindsight-like behavior by monkeys.^59–62^ In these approaches, monkeys are rewarded for locating a visual target, but on trials in which no target is presented, monkeys are rewarded for taking an “opt-out” action. Choosing to opt out is interpreted as the monkey not seeing any target on that trial. Monkeys learn to locate the target when it is present and opt out in its absence. After unilateral removal of V1, monkeys opt out even when the target *is* present in the contralateral visual field, indicating that they are blind to the target (i.e., exhibit blindness). These blind monkeys might have no sight, lacking both conscious and unconscious vision, or they might only lack conscious vision, possessing blindsight. Strikingly, when the investigators remove the opt-out option, the monkeys locate the target, consistent with blindsight. For brevity, we will call what an animal loses in a model of blindsight “conscious vision” and what remains “unconscious vision,” but without direct access to the animal’s subjective experience, we must consider both putative.

While there is unique value in studying monkeys, the toolkit for neural manipulation and the scale of experiments are limited as compared with mice. Prior work has demonstrated that rodents can detect and discriminate visual stimuli, and, like primates, removal,^63–77^ ablation,^78–80^ and suppression^73–77,81–88^ of V1 in rodents reduces contrast sensitivity and reduces or eliminates discrimination capability. It has been unclear whether conscious vision or blindsight accounts for the visual behavior that remains after V1 damage. Here, we developed the first mouse model of blindsight that allowed us to explore whether mice lose conscious vision and rely on blindsight after brain damage. After training separate groups of mice on a novel visual task, we employed three complementary methods for disabling V1 and the hippocampus to investigate effects on vision. We used mechanical removal by suction and excitotoxic ablation by ibotenate injection to examine vision during the chronic phase, and we used reversible neural suppression by muscimol to examine vision during the acute phase. Our results outline a new model for conscious visual perception in which V1 and the hippocampus operate in series to cause conscious vision in the intact brain, but when either is permanently damaged, each of these two brain areas can compensate for the loss of the other.

## RESULTS

### Unilateral removal of the primary visual cortex (V1) results in contralateral blindness

The first step to show blindsight-like behavior by mice is to show that mice act blind after damage to V1, as humans do.^20^ Prior work with rodents did not observe substantial blindness after removal of V1, as these rodents could still detect high-contrast visual stimuli.^63–73,76,77^ This raises the question of whether mice do not need V1 for normal vision or whether, like primates, they lose conscious vision after removal of V1 and use blindsight to maintain good performance.

**Figure 1.**
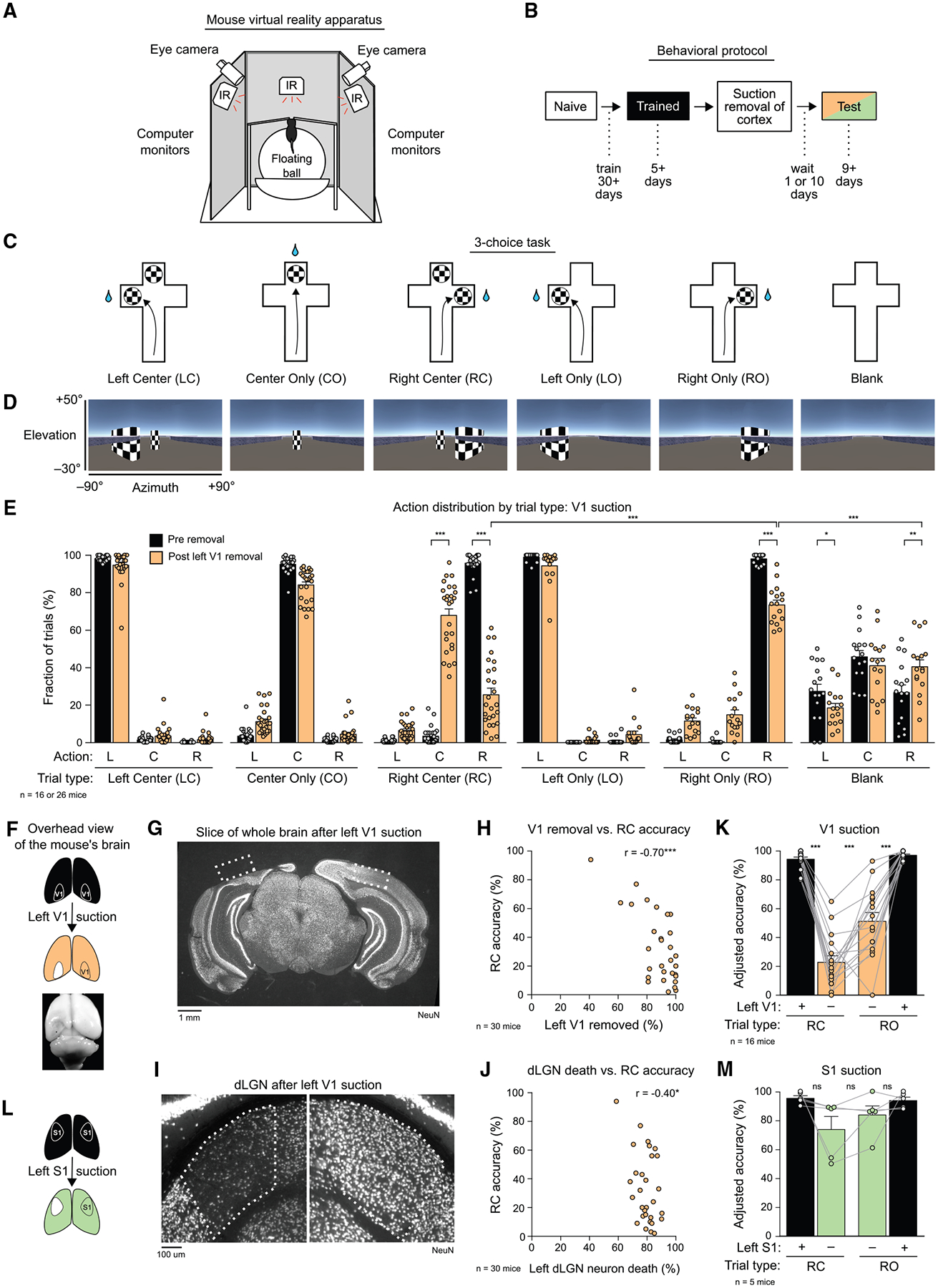
Mice exhibit blindness with blindsight-like behavior after unilateral removal of V1 (A) Virtual reality apparatus with binocular eye tracking. IR, infrared LED array. (B) Behavioral training protocol. We trained mice prior to removal of left V1 or left S1 and tested behavior the next day or 10 days after surgery. Color of data in subsequent panels corresponds to data from the protocol phase with the same color. (C) Top-down view of trial types of the 3-choice task. Arrow and water droplet indicate rewarded action. Mice start at the bottom arm and must navigate to one of the three top locations to finish each trial. (D) 1^st^-person view of the start of each trial type. Checkerboard targets are visible below ground level, as if the mouse is running on a clear surface. Walls are short, gray, and visible just below the horizon. Small white text presenting real-time statistics was present at the top center of the display. We removed this for clarity here and in all subsequent 1^st^-person views. (E) Actions of mice before (black) and after (orange) removal of left V1. Following removal, mice exhibit blindness by running to the unrewarded central target on RC trials and exhibit blindsight-like behavior by running right on RO trials more often than on RC and blank trials. L, left; C, center; R, right action. *n* = 16–26 mice. (F) Top: schematic of V1 surgical removal. Bottom micrograph: dorsal view of a mouse brain after removal of left V1. (G) Coronal section showing the removal of left V1, with an outline demarcating where left V1 should be and where right V1 is. Fluorescence is an anti-NeuN stain. (H) Left V1 removal correlates with blindness on RC trials. *n* = 30 mice. (I) Coronal section showing degeneration in left dLGN following left V1 removal, with an outline demarcating dLGN. Fluorescence is an anti-NeuN stain. This mouse was perfused 7 weeks after V1 aspiration. (J) Left dLGN degeneration after V1 removal correlates with blindness on RC trials. *n* = 30 mice. (K) Mice exhibit right hemifield blindness after left V1 removal, as demonstrated by a drop in RC-adjusted accuracy. Mice exhibit right hemifield blindsight-like behavior after left V1 removal, as demonstrated by higher RO-adjusted accuracy relative to RC-adjusted accuracy. *n* = 16 mice. (L) Schematic of left somatosensory cortex (S1) removal. (M) Removal of S1 does not have a significant effect on vision on either RC or RO trials. *n* = 5 mice. Each dot is one mouse. Error bars are SEM. *p < 0.05, **p < 0.01, ***p < 0.001; ns, *p* ≥ 0.05; paired *t* test comparing indicated groups (E, K, and L) or slope of linear regression (H and J). See also Video S1 and Figures S1–S4.

To test whether mice rely on V1 for normal vision, we trained head-fixed mice to run to large, high-contrast, black-and-white targets in a 3-choice, self-paced virtual reality navigation task (Figure 1A and the left three columns in Figures 1C and 1D). On left-center (LC) trials, a target appears on the left and in the center, and mice receive a reward for running to the left target. On right-center (RC) trials, a target appears on the right and in the center, and mice receive a reward for running to the right target. In center-only (CO) trials, a target only appears in the center, and mice receive a reward for running to the central target. With this training paradigm, the central target represents the opt-out option—the choice the mouse should make when it does not detect the left and right targets. Crucially, CO trials enable assessment of whether a mouse is blind following an alteration of the brain because if the altered mouse consistently runs to the unrewarded central target on LC or RC trials, such behavior would be consistent with the mouse only seeing the central but not the lateral target.

Mice learned this 3-choice task well, performing with a mean accuracy exceeding 95% on each trial type (Figure 1E, black bars). We then aspirated the left V1 (Figures 1F and 1G) and waited one or 10 days before assessing behavior (Figure 1B). The effect of suction was similar regardless of the delay (Figures S1A and S1B), so we pooled the results from both experiments. Strikingly, removal of left V1 caused mice to run primarily to the unrewarded central target on RC trials, consistent with being blind in the right visual field (Figure 1E, orange bars; Video S1). Mice completed correct trials faster than incorrect trials (Figure S1C), and mice ran to the central target on RC trials faster after removal of left V1 than before removal (Figure S1D).

To measure the completeness of V1 removal, we conducted rigorous quantitative histological analysis to identify V1 and neighboring areas in brain sections (STAR Methods; Figure S2). If we removed less than 80% of left V1, we excluded the mouse’s behavior from analysis (4 of 30 mice were excluded). After exclusion, we removed 89% of left V1 brain tissue on average. The extent of removal of left V1 was strongly negatively correlated with accuracy on RC trials (Figure 1H). Suction also removed areas neighboring V1, but removal of V1 had the largest negative correlation with RC accuracy (Figures S1E–S1O). In addition, regression models with RC accuracy as the dependent variable and with two independent variables, one of which was the size of removal of V1 and the other was the size of removal of each of the neighboring areas, always yielded a larger and more significant coefficient for V1 than for any other brain area. Altogether, we conclude that it is the removal of V1 and not an adjacent area that is most predictive of the drop in RC accuracy.

Surgical removal of V1 is known to cause retrograde degeneration of LGN, its principal source of visual input, in humans,^89,90^ monkeys,^61,91^ and rodents.^79,92^ We quantified this degeneration in humans with scotoma and found that the LGN ipsilateral to the damaged V1 shrank by 34% on average (Figure S3). The fact that two brain areas are compromised after V1 removal introduces ambiguity: the loss of conscious vision might be due to (1) the direct loss of V1, (2) the indirect damage to dLGN, or (3) the combined effect on both areas. Mouse dLGN also degenerated after V1 removal (Figure 1I). The fraction of neurons killed in mouse dLGN negatively correlated with RC accuracy (Figure 1J). In fact, selective ablation of mouse V1 by a procedure that leaves dLGN intact did not result in blindness during the chronic phase following injury (see below), suggesting that both V1 and dLGN must be damaged to cause a loss of conscious vision.

### Blindsight-like behavior on a detection task after unilateral V1 removal

Having shown that removal of V1 causes blindness in mice, we next asked whether these blind mice could use blindsight to maintain good behavioral performance. To distinguish between blindsight and no sight, we introduced three new trial types during training prior to V1 removal (the right three columns of Figures 1C and 1D): left-only (LO) trials in which only the left target appears and provides reward, right-only (RO) trials in which only the right target appears and provides reward, and blank trials in which no target appears and no action provides reward. By accounting for eye movements and restricting head movements through head fixation, mice could not “cheat” by moving the right target into the left visual hemifield (Figure S4).

Removing the central target on RC and LC trials to create RO and LO trials, respectively, might cause a blind mouse to behave as if the opt out were absent. Without the opt out, mice might now use the “unseen” visual target to guide their behavior, as observed with blindsight by humans and monkeys. If this were true, then a left V1-removed mouse without conscious vision but with blindsight would incorrectly run to the center target on RC trials but correctly run right on RO trials. Blank trials also lack the central target, but a blindsighted mouse would run less often to the right on blank trials than on RO trials, because blank trials lack the right target to guide the mouse’s behavior.

Consistent with blindsight, left V1-removed mice ran to the right on RO trials more often than on RC trials (Figure 1E). In support of the right target guiding the mouse’s behavior on RO trials (rather than the absence of the left and central targets), mice ran to the right more often on RO trials than on blank trials, and the overall action distributions on RO trials were very different from blank trials (Figure 1E; *p* < 0.001, χ^2^ test). Like these mice, monkeys with unilateral removal of V1 also behaved differently on blank trials than on trials in which the stimulus was present solely in the contralateral hemifield.^93^

### Blindsight-like behavior by individual mice as well as by the group

In humans, blindsight is diagnosed in each individual patient, so we determined whether individual mice exhibited blindsight-like behavior. The frequency of running to the right on CO and blank trials reflects each individual mouse’s right bias in the absence of a right target. To calculate how much the visual target on the right drove the behavioral decision to run to the right, we deducted these right biases when calculating two new metrics, RC-adjusted accuracy and RO-adjusted accuracy, respectively (STAR Methods). We defined a mouse as blind if their RC-adjusted accuracy was less than 50% and designated a blind mouse as having blindsight-like behavior if their RO-adjusted accuracy was statistically significantly higher than their RC-adjusted accuracy. Using these criteria, 88% of V1-removed mice were blind (14 of 16), and 57% of the blind mice exhibited blindsight-like behavior (8 of 14; Figure 1K), in line with the frequency of blindsight in humans.^25,26,30^ Furthermore, V1-removed mice exhibited blindsight-like behavior at the group level (Figure 1K). This effect was specific to V1, as removal of the left primary somatosensory cortex (S1) did not induce blindness at either the individual or group level (Figures 1L and 1M).

### Blindsight-like behavior is the best interpretation of the effect of V1 removal

We considered three alternative explanations for the mouse’s behavior after V1 removal that do not depend on the mouse losing conscious vision. First, removal of left V1 might cause a reduction of salience in the right visual field without abolishing conscious vision. A visual cue can activate V1 and enhance spatial attention at the cued location,^94^ suggesting that V1 supports salience-driven bottom-up attention. A field-specific drop in salience might cause mice to ignore the right target when there is a competing central target on RC trials but notice the right target when it appears alone on RO trials. To test whether removal of left V1 causes a loss of salience in the right visual field, we simulated such a loss by reducing the salience of stimuli presented to intact mice and compared their behavior with that of V1-removed mice.

**Figure 2.**
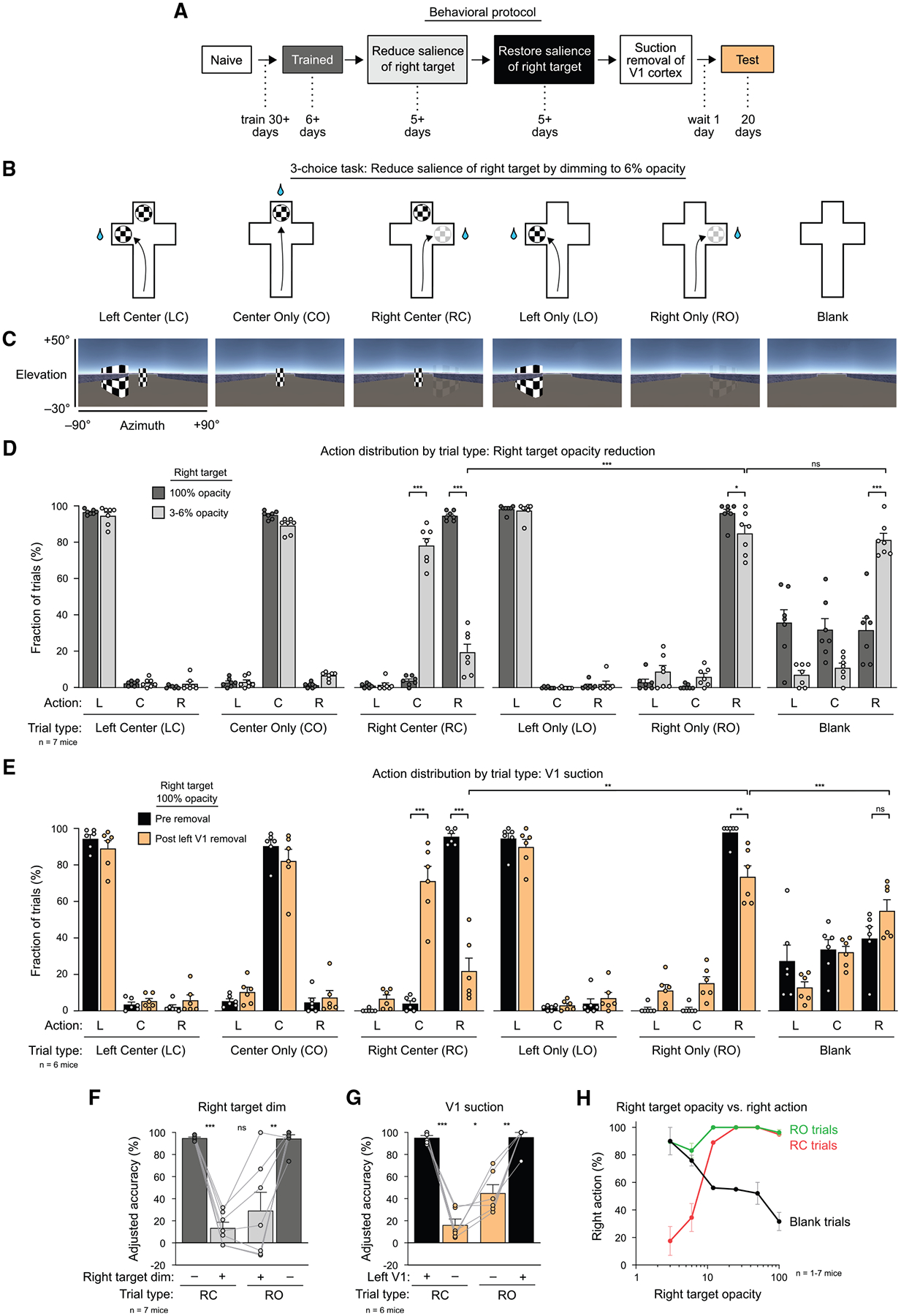
Reducing the salience of the contralateral target does not have the same behavioral effect as unilateral removal of V1 (A) Behavioral protocol outlining the different phases of the experiment, with fill color corresponding to data shown below. (B) Top-down view of trial types of the 3-choice task with right targets of reduced salience (6% opacity), corresponding to the third phase of the behavioral protocol (lightest gray). Arrow and water droplet indicate rewarded action. Mice start at the bottom arm and must navigate to one of the three top locations to finish each trial. (C) 1^st^-person view of the start of each trial type. The right target is set to 6% opacity and appears less salient than the other targets. (D) Actions of intact mice with the right target at 100% opacity (dark gray) and 3%–6% opacity (light gray). At low opacity, intact mice run to the central target on RC trials, and they run right on RO trials at the same rate as they run right on blank trials, indicating a failure to distinguish between RO and blank trials. This failure does not occur after V1 removal (E). *n* = 7 mice. (E) Actions of the same mice as in (D) with the right target at 100% opacity before (black) and after (orange) left V1 removal. V1-removed mice run to the central target on RC trials, and they run right on RO trials at a higher rate than they run right on blank trials, indicating that they can distinguish between RO and blank trials. *n* = 6 mice. (F) When the right target is less salient, RO-adjusted accuracy is not higher than RC-adjusted accuracy in intact mice. *n* = 7 mice. (G) When we remove left V1, RO-adjusted accuracy is higher than RC-adjusted accuracy, consistent with blindsight. *n* = 6 mice. (H) The rate of right actions on RC and RO trials as the right target’s opacity changes. Starting at 100% opacity, we lowered the opacity until mice ran to the central target on RC trials, which occurred at 6% or 3% opacity (red line). Mice continue to run right on these low-opacity RO trials (green line), though the rate of right action is equal to that of blank trials (black line), indicating that mice could not distinguish between low-opacity right and blank trials. *n* = 1–7 mice. For the bar graphs, each dot is one mouse. Error bars are SEM. *p < 0.05, **p < 0.01, ***p < 0.001; ns, *p* ≥ 0.05; paired *t* test comparing indicated groups. L, left; C, center; R, right action. See also Figures S5 and S6.

We trained a new group of mice on the 3-choice task and then lowered the opacity of the right target until mice ran to the central target on RC trials (dim-RC; Figures 2A–2D and 2H), as left V1-removed mice do. Consistent with what we observed after V1 removal, these mice also ran to the right on dim-RO trials more than on dim-RC trials (Figure 2D). In contrast to V1-removed mice, however, these mice failed to run to the right more often on dim-RO trials than on blank trials (Figure 2D), suggesting that they were unable to distinguish between the presence and absence of a solo right target. When we restored the opacity of the right target to 100% and then removed left V1, these mice were able to distinguish between the presence and absence of a solo right target (Figure 2E), replicating the previous result (Figure 1E). Put another way, adjusted accuracy on RC and RO trials was statistically equal when the right target had low opacity, whereas RO-adjusted accuracy was consistently greater than RC-adjusted accuracy after we restored opacity to 100% and then removed left V1 (Figures 2F and 2G). Therefore, although removal of V1 likely causes a loss of salience (i.e., objects in a blind field are less salient than those in a non-blind field), a loss of salience alone does not, in the conditions tested here, completely recapitulate what happens after V1 removal, even in the exact same mice. Thus, we consider the behavior observed after V1 removal as inconsistent with or greater than the behavioral effect of a loss of salience. Monkeys also largely retained salience processing after V1 removal.^95^

A second alternative interpretation is that removal of V1 might cause another kind of attentional defect, such as visual extinction^96–98^ or neglect,^97,99^ while preserving conscious vision. We consider this interpretation poorly supported for the following reasons. First, damage to brain areas other than V1 in humans, such as the temporo-parietal junction^98^ and the temporal lobe,^100^ causes visual extinction and neglect, respectively. Second, neglect causes patients to over-explore the visual space ipsilateral to the brain damage.^101^ Yet following the removal of left V1, mice did not over-explore space ipsilateral to the damage, demonstrated by their (1) running to the left on blank trials less often (Figure 1E) and (2) running without a trajectory shift to the left on CO trials (Figures S5A–S5F). Instead, these mice over-explored contralateral space as V1-damaged patients with hemianopia do,^101^ demonstrated by their (1) running to the right on blank trials more often (Figure 1E) and (2) running with a trajectory shift to the right on CO trials (Figures S5A–S5F).

To test whether a mouse with neglect-like behavior would run to the central target on RC trials and right on RO trials, we used ibotenate to pharmacologically ablate the left SC, which, when damaged, can induce neglect in humans^102^ and neglect-like behavior in monkeys.^103^ Consistent with neglect, these mice exhibited a strong leftward (ipsiversive) bias on CO, RC, and RO trials (Figure S6). Since neglect-like behavior did not match the behavior we observed after V1 removal, V1-removed mice are unlikely to be exhibiting neglect.

A third alternative interpretation is that when a mouse opts out of a trial by running to the central target on RC trials, that action could indicate that the mouse is uncertain of whether a right target is present rather than indicating that the mouse does not see the right target. Rats and mice have been trained to use opt-out responses to indicate such a lack of confidence.^104–106^ Such an interpretation is not viable for our results for several reasons. First, in the prior work, rats were rewarded for opting out, which does not occur here when a mouse runs to the central target on RC trials. Second, in the prior work, rats could skip a difficult trial by opting out, which does not occur here, as incorrect trials have a longer delay than correct ones. Third, in the prior work, rats performed equally or worse when they were not allowed to opt out, whereas our V1-removed mice performed better without the opt out, as seen on RO trials as compared with RC trials. For these reasons, we do not favor the interpretation that running to the central target on RC trials indicates a mouse’s uncertainty.

Altogether, we conclude that the best interpretation consistent with the behavioral results is that left V1-removed mice lose conscious vision in their right field and use a blindsight-like ability to run correctly to the right target on RO trials.

### Blindsight-like behavior on a localization task after unilateral V1 removal

**Figure 3.**
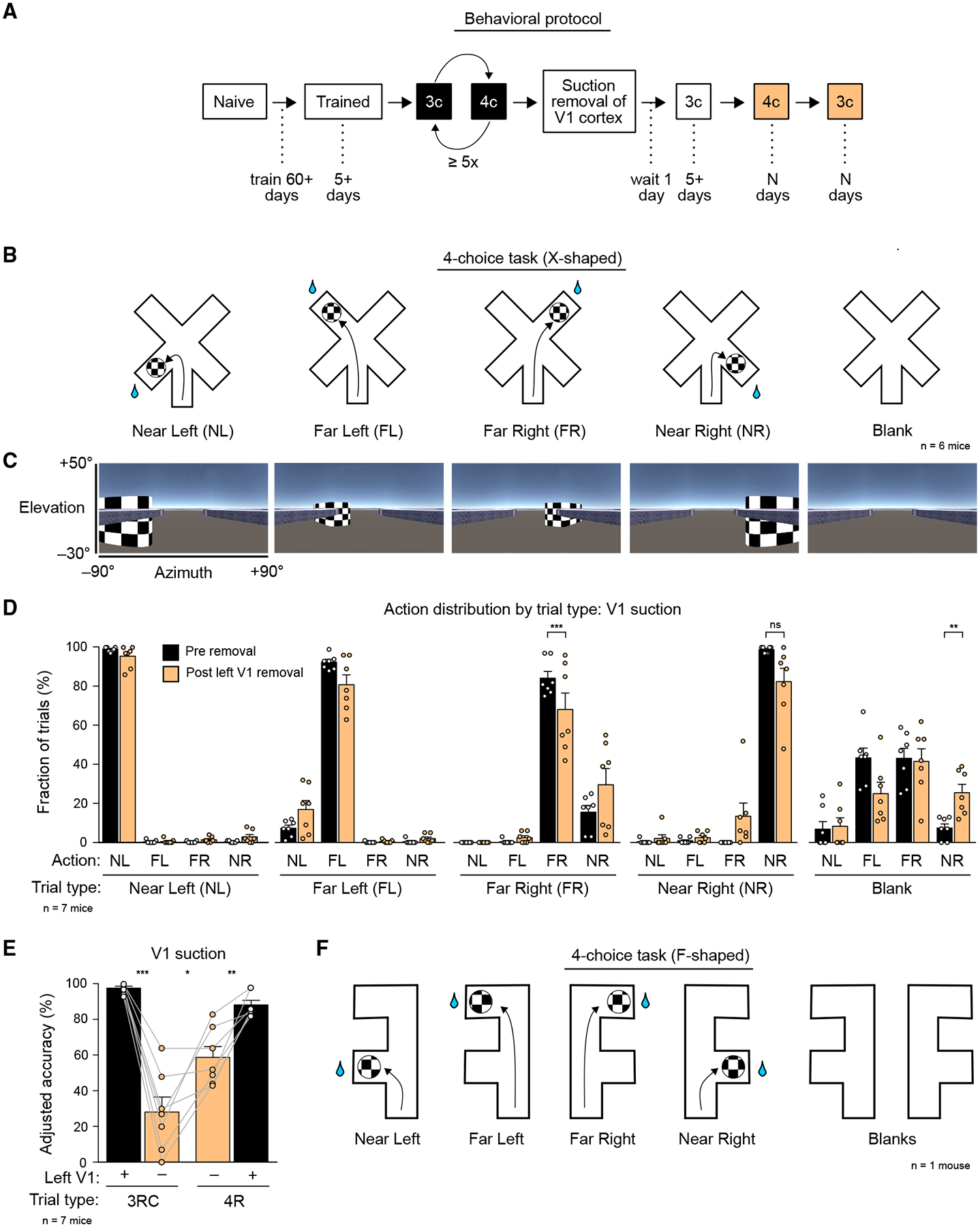
Mice exhibit blindsight-like behavior after unilateral removal of V1 on a 4-choice localization task (A) Behavioral training protocol. We trained mice on both 3- and 4-choice tasks prior to removal of left V1, with each task trained on alternate days for at least five replications before surgery. Following surgery, we tested mice on the 3-choice task for five or more days, followed by N days on the 4-choice task until behavior was accurate, and finally switched back to the 3-choice task for the same number of N days to test for blindness on RC trials. 3c, 3-choice; 4c, 4-choice. Shading color matches the data presented below. (B) Top-down view of trial types of the 4-choice task. Arrow and water droplet indicate rewarded action. Mice start at the bottom and must navigate to one of the four locations to finish each trial. (C) 1^st^-person view of the start of each trial type. (D) Actions of mice before (black) and after (orange) removal of left V1. Following removal, mice exhibited blindsight-like behavior by showing above-chance accuracy on FR and NR trials. *n* = 7 mice. (E) Removal of left V1 results in right hemifield blindness, as measured on the second set of 3-choice RC trials following surgery, and blindsight-like behavior, as demonstrated by an increase in adjusted accuracy on 4-choice right (4R) trials relative to RC-adjusted accuracy. *n* = 7 mice. (F) Alternative 4-choice design. One mouse failed to master the standard version of the 4-choice task (B) and instead mastered this alternative. (D) and (E) pool the results from the X-shaped and F-shaped designs. Each dot is one mouse. Error bars are SEM. *p < 0.05, **p < 0.01, ***p < 0.001; ns, *p* ≥ 0.05; paired *t* test comparing indicated groups.

Although the 3-choice task shows that a target in a mouse’s blind field can nonetheless guide their behavior, this does not demonstrate that mice can accurately locate a target within their blind field, as shown by humans^21,24^ and monkeys.^59,62^ Therefore, we implemented a 4-choice task in which the mouse must locate a target as either near or far within the same visual hemifield and without an opt out. We trained a subset of the mice above (from Figure 1E) on both the 3-choice and 4-choice tasks prior to left V1 removal (Figure 3A). In the 4-choice task, mice learn to run to a single target presented in one of four locations: near left (NL), far left (FL), far right (FR), and near right (NR) (Figures 3B, 3C, and 3F). We also included blank trials to measure the actions taken in the absence of a target. If a left V1-removed mouse acts blind on RC trials in the 3-choice task but successfully locates the right target in the 4-choice task, then their behavior would be consistent with blindsight.

Consistent with blindsight, removal of left V1 had no effect on the accuracy of locating NR targets, while there was a small but significant reduction in locating FR targets (Figure 3D). Near targets were easier to navigate to than far targets, as a misnavigation to the far target could result in a mouse unintentionally running to the near target in front of it. Of the seven mice trained on both the 3- and 4-choice tasks, six were blind on RC trials in the 3-choice task following removal of left V1. 67% of these blind mice (4 of 6) performed significantly better on 4-choice right (4R) trials as compared with RC trials (Figure 3E), consistent with blindsight in individual mice. Furthermore, these mice exhibited blindsight-like behavior at the group level (Figure 3E). Overall, the results of the 4-choice task demonstrate blindsight-like localization, lending further support to the 3-choice task as a valid assay for blindsight.

### Ablation of V1 by ibotenate causes limited dLGN degeneration

**Figure 4.**
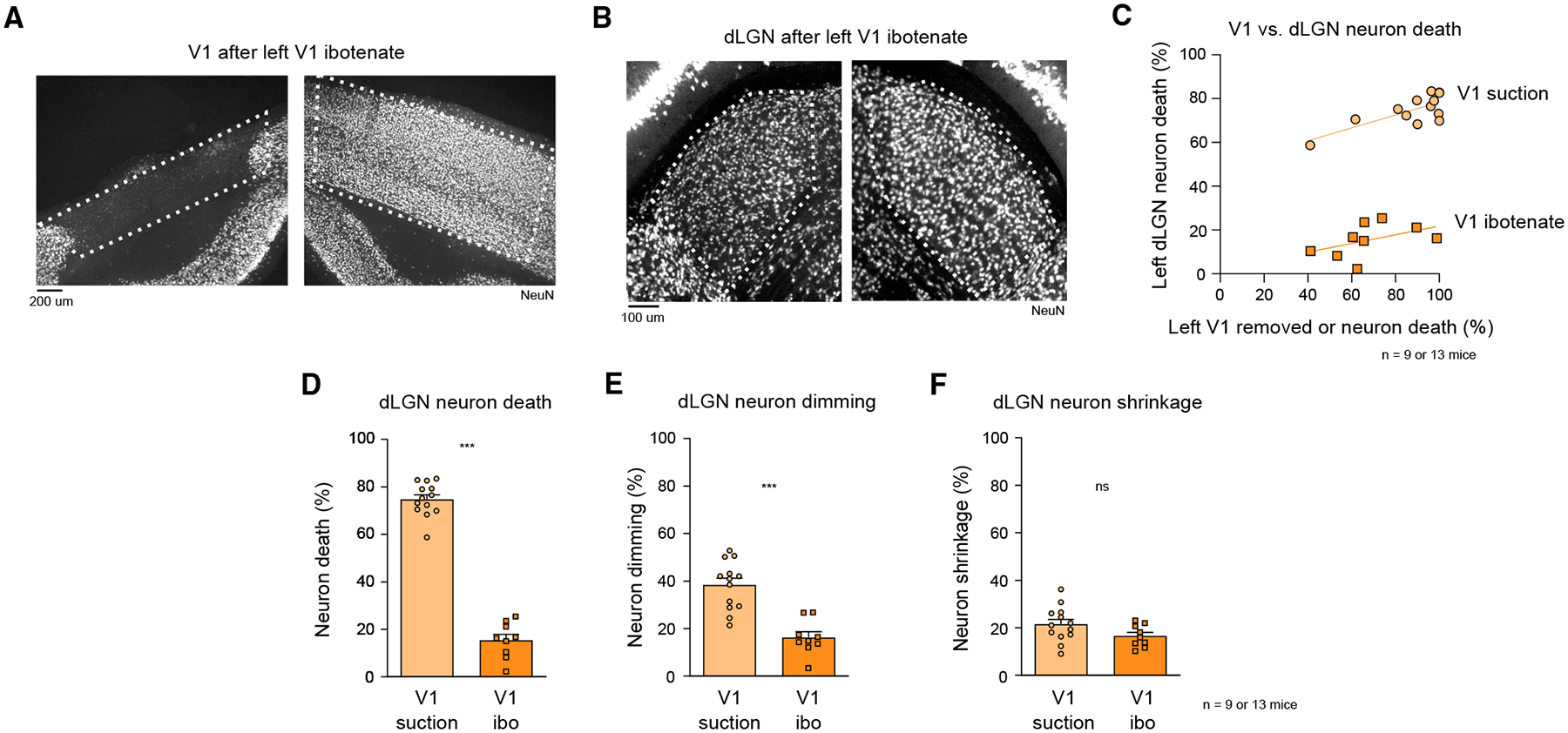
Ablation of V1 by ibotenate causes limited dLGN degeneration (A) Coronal section showing left V1 ablation by ibotenate injection into left V1, with outlines demarcating V1. Fluorescence is an anti-NeuN stain. (B) Coronal section showing limited degeneration in left dLGN following left V1 ablation by ibotenate, with outlines demarcating dLGN. Same mouse as in (A). Fluorescence is an anti-NeuN stain. This mouse was perfused 7 weeks after ibotenate injection. (C) As the damage to V1 varies in size, V1 suction (circles, orange regression line, *p* = 0.004) results in larger death in dLGN than V1 ablation by ibotenate (squares, bright orange regression line, *p* = 0.21). (D) V1 ibotenate causes less death of dLGN neurons than V1 suction. (E) V1 ibotenate causes less dimming of anti-NeuN stain in dLGN neurons than V1 suction. (F) Both V1 suction and ibotenate cause surviving ipsilateral dLGN neurons to be 20% smaller than those in contralateral dLGN. (C–F) V1 suction, *n* = 13 mice; V1 ibotenate, *n* = 9 mice. Each dot is one mouse. Error bars are SEM. **p < 0.01, ***p < 0.001; ns, *p* ≥ 0.05; unpaired *t* test comparing indicated groups.

Since V1 removal results in substantial dLGN degeneration, we investigated whether the loss of conscious vision is due to damage to V1, the consequent degeneration of dLGN, or both. Since aspiration of V1 might cause dLGN degeneration by cutting dLGN axons, we tested whether injection of ibotenate (ibotenic acid), a method of ablation that spares fibers of passage,^107^ would not cause dLGN degeneration. We trained mice on the 3-choice task, injected ibotenate into the left V1 to cause an ablation, tested their behavior (reported below), and then perfused the mice to examine dLGN. Indeed, ablation of V1 by ibotenate caused much less neuronal death in dLGN compared with V1 removal (Figures 4A–4D). Surviving dLGN neurons stained less intensely with NeuN antibodies after V1 suction than ibotenate ablation (Figure 4E), though they appeared dimmer under both conditions when compared with the intact dLGN on the right side. Finally, both V1 suction and ibotenate caused the surviving dLGN neurons to be equally smaller than those in the intact dLGN on the right side (Figure 4F). The V1 suction mice shown here are a subset of those trained on the 3-choice task reported above (Figure 1; STAR Methods).

### Selective unilateral disruption of V1 causes no sight acutely, but normal vision returns chronically

**Figure 5.**
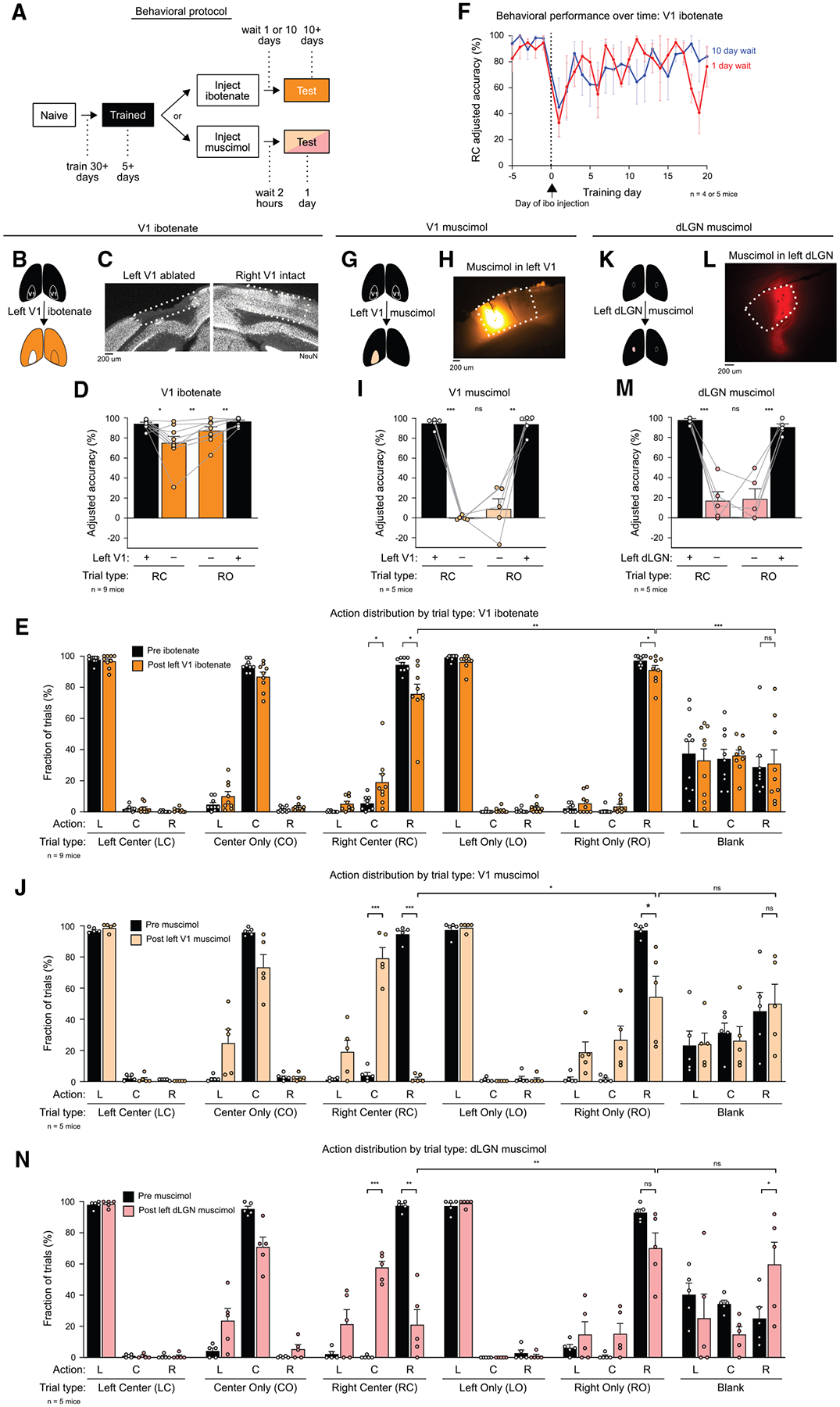
Selective unilateral disruption of V1 causes no sight acutely, but normal vision returns chronically (A) Behavioral training protocol. Trained mice were either injected with ibotenate and tested behaviorally 1 or 10 days after surgery or injected with muscimol and tested behaviorally approximately 2 h after surgery. (B) Schematic of ibotenate ablation of left V1. (C) Coronal section showing damage to left V1. V1 is outlined with a white dotted line. Fluorescence is an anti-NeuN stain. (D) V1 ablation by ibotenate does not cause blindness. *n* = 9 mice. (E) Actions of mice before (black) and after (bright orange) ibotenate ablation of left V1. Following ablation, mice exhibit modest reductions in RC and RO accuracies, but not enough to be classified as blind. *n* = 9 mice. (F) Mice tested 1 or 10 days after ibotenate injection into V1 do not have different RC-adjusted accuracies. 1-day wait, *n* = 4 mice; 10-day wait, *n* = 5 mice. (G) Schematic of suppressing left V1 with muscimol. (H) False-colored coronal section showing the spread of fluorescence-tagged muscimol primarily in V1, outlined with a white dotted line. (I) Suppressing V1 with muscimol causes no sight. *n* = 5 mice. (J) Actions of mice before (black) and after (light orange) muscimol suppression of left V1. Following suppression, mice exhibit profound blindness without blindsight-like behavior. *n* = 5 mice. (K) Schematic of suppressing left dLGN with muscimol. (L) False-colored coronal section showing spread of fluorescence-tagged muscimol primarily in dLGN, outlined with a white dotted line. (M) Suppressing dLGN with muscimol causes no sight. *n* = 5 mice. (N) Actions of mice before (black) and after (pink) muscimol suppression of left dLGN. Following suppression, mice exhibit strong blindness without blindsight. *n* = 5 mice. Each dot is one mouse. Error bars are SEM. *p < 0.05, **p < 0.01, ***p < 0.001; ns, *p* ≥ 0.05; paired *t* test comparing indicated groups. (E, J, and N) L, left; C, center; R, right action. See also Figure S7.

Having identified a V1 ablation technique that spares dLGN histologically, we injected ibotenate into the left V1 of trained mice (Figure 5A, top path). The effect of ibotenate was similar whether mice received a 1- or 10-day break after surgery (Figure 5F), so we pooled the results. Unexpectedly, ablation of left V1 by ibotenate caused a very small reduction in RC accuracy, unlike aspiration (Figures 5B–5E and S7). This raised the possibility that the loss of conscious vision caused by V1 removal is best explained by the degeneration of dLGN or by the combined damage to both areas.

The results of ibotenate ablation of V1 are consistent with either (1) V1 being dispensable for conscious vision or (2) V1 being important for conscious vision but its chronic loss causing compensation to occur. To distinguish between these interpretations, we used neural suppression by muscimol to silence V1 and acutely examine behavior 2 h post-injection (Figure 5A, bottom path). Suppressing left V1 using muscimol induced no sight in mice, acting blind without blindsight-like behavior (Figures 5G–5J). Prior studies that suppressed V1 in humans^108^ and monkeys^56^ also observed no sight. Suppressing the left dLGN, like suppressing V1, also caused no sight (Figures 5K–5N), consistent with results from monkeys.^29,109,110^ Suppressing a brain area ventromedially adjacent to dLGN, the somatosensory thalamus (VP), had no effect on vision (data not shown). Across species, these results suggest that both V1 and dLGN are necessary for conscious vision in the intact brain. They also suggest that the minor effect on conscious vision by ibotenate ablation of V1 might be due to an eventual compensation by an as-yet-unidentified brain area.

### Unilateral disruption of the dorsal hippocampus causes blindsight-like behavior

**Figure 6.**
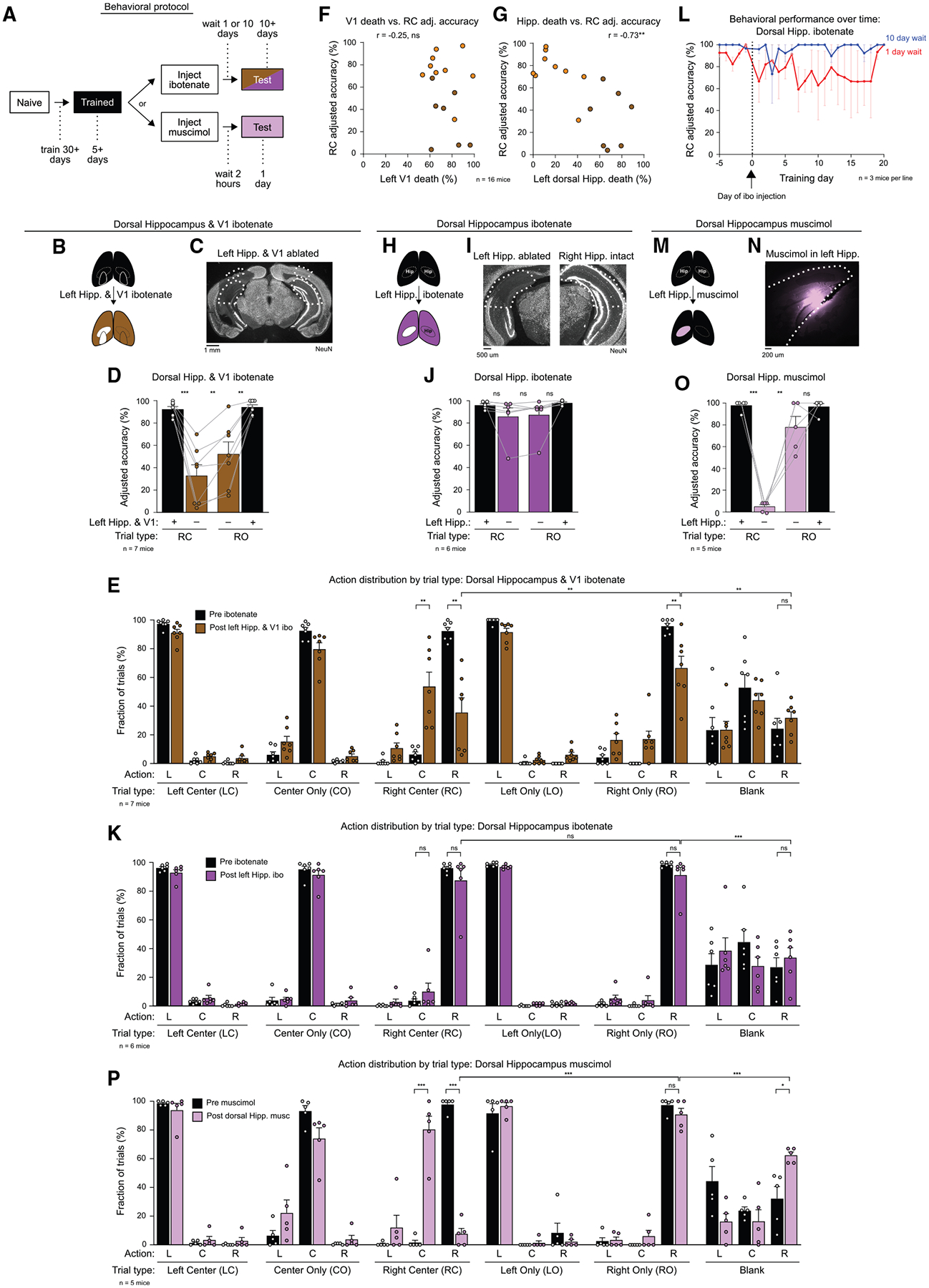
Unilateral disruption of the dorsal hippocampus causes blindsight-like behavior (A) Behavioral training protocol. Trained mice were either injected with ibotenate and tested behaviorally 1 or 10 days after surgery or injected with muscimol and tested behaviorally approximately 2 h after surgery. (B) Schematic of ibotenate ablation of the left dorsal hippocampus and left V1. (C) Coronal section showing damage to the left dorsal hippocampus and left V1. Top left-right pair of dotted areas demarcates V1, and bottom left-right pair demarcates the dorsal hippocampus. Fluorescence is an anti-NeuN stain. (D) Ablation of the left dorsal hippocampus and left V1 by ibotenate causes blindsight-like behavior. *n* = 7 mice. (E) Actions of mice before (black) and after (brown) ibotenate ablation of the left dorsal hippocampus and left V1. Following ablation, mice exhibit blindsight-like behavior. *n* = 7 mice. (F) The extent of left V1 ablation by ibotenate does not correlate with RC-adjusted accuracy. (G) The extent of left dorsal hippocampus ablation by ibotenate negatively correlates with RC-adjusted accuracy when V1 is also ablated. (F and G) Bright orange dots show mice categorized as V1-only ablations and shown in Figure 5D, whereas dark brown dots show mice categorized as dual V1-and hippocampus-ablations and shown in (D) above. *n* = 16 mice. (H) Schematic of ibotenate ablation of the left dorsal hippocampus only. (I) Coronal section showing damage to the left dorsal hippocampus. Dotted areas demarcate the left and right hippocampus. Fluorescence is an anti-NeuN stain. (J) Ablation of the dorsal hippocampus by ibotenate does not cause blindness. *n* = 6 mice. (K) Actions of mice before (black) and after (purple) ibotenate ablation of the left dorsal hippocampus. Ablation does not affect behavior. *n* = 6 mice. (L) Mice tested 1 or 10 days after ibotenate injection into the dorsal hippocampus do not have different RC-adjusted accuracies. 1-day wait, *n* = 3 mice; 10-day wait, *n* = 3 mice. (M) Schematic of muscimol suppression of the left dorsal hippocampus. (N) False-colored coronal section showing spread of fluorescence-tagged muscimol primarily in the left dorsal hippocampus, outlined with a dotted line. (O) Suppressing the left dorsal hippocampus with muscimol causes blindsight-like behavior. *n* = 5 mice. (P) Actions of mice before (black) and after (light purple) muscimol suppression of the left dorsal hippocampus. Suppressing the hippocampus causes the most profound blindsight-like behavior out of all manipulations. *n* = 5 mice. Each dot is one mouse. Error bars are SEM. *p < 0.05, **p < 0.01, ***p < 0.001; ns, *p* ≥ 0.05; paired *t* test comparing indicated groups (bar charts) or slope of linear regression (scatterplots).

Since the hippocampus sits at the top of the visual cortical hierarchy^52^ and is necessary for the formation of conscious memories^48^ and visual imagination,^49^ we hypothesized that the hippocampus would be important for conscious vision. If true, then the hippocampus might be the brain area that compensates for the loss of V1 after ibotenate ablation. Therefore, we ablated both left V1 and the left dorsal hippocampus with ibotenate and tested behavior 10 days post-injection (Figure 6A, top path). Strikingly, these double-ablated mice exhibited blindness with blindsight-like behavior (Figures 6B–6E). At the individual level, 71% of dual-ablated mice (5 of 7) were blind, and 40% of the blind mice (2 of 5) exhibited blindsight-like behavior. Whereas the size of V1 ablation did not correlate with RC-adjusted accuracy in these mice (Figure 6F), in the presence of a V1 ablation, the extent of dorsal hippocampal ablation correlated remarkably well with RC-adjusted accuracy (Figure 6G). Importantly, ibotenate ablation of the left hippocampus alone had no effect on vision (Figures 6H–6K). The behavioral effect of single hippocampal ablation was similar regardless of whether we waited one or 10 days before behavioral testing (Figure 6L), so we pooled the results from both experiments. Taken together, these results suggest that the hippocampus can compensate for the loss of V1 to restore vision during the chronic phase following injury.

To assess the role of the hippocampus in vision in the intact brain, we suppressed the left hippocampus using muscimol, waited 2 h, and then tested vision. If the hippocampus operates downstream of V1 in the intact brain for conscious vision, then suppressing it with muscimol would likewise lead to blindness. Consistent with this hypothesis, suppressing the left dorsal hippocampus caused right-sided blindness, but, unlike suppressing V1, it revealed blindsight-like behavior (Figures 6M–6P). Hippocampal suppression had a weaker effect on vision than suppressing the directly dorsal (V1) and ventral (dLGN) brain areas (Figures 5G–5N), suggesting that the loss of conscious vision and blindsight-like behavior observed after hippocampal suppression was not due to leakage of muscimol into these adjacent brain areas.

As described above for removal of V1, we interpret this behavior as more likely to be hemianopia with blindsight than neglect. Mice with suppression of the left hippocampus by muscimol over-explored space contralateral to the suppression, consistent with the exploration behavior of human hemianopes.^101^ These mice demonstrated this in two ways: (1) they ran to the location on the right on blank trials more often following hippocampal suppression (Figure 6P), and (2) their running trajectories in the 3-choice task shifted to the right on CO trials following hippocampal suppression (Figures S5G–S5J).

### The hippocampus responds to drifting visual gratings

**Figure 7.**
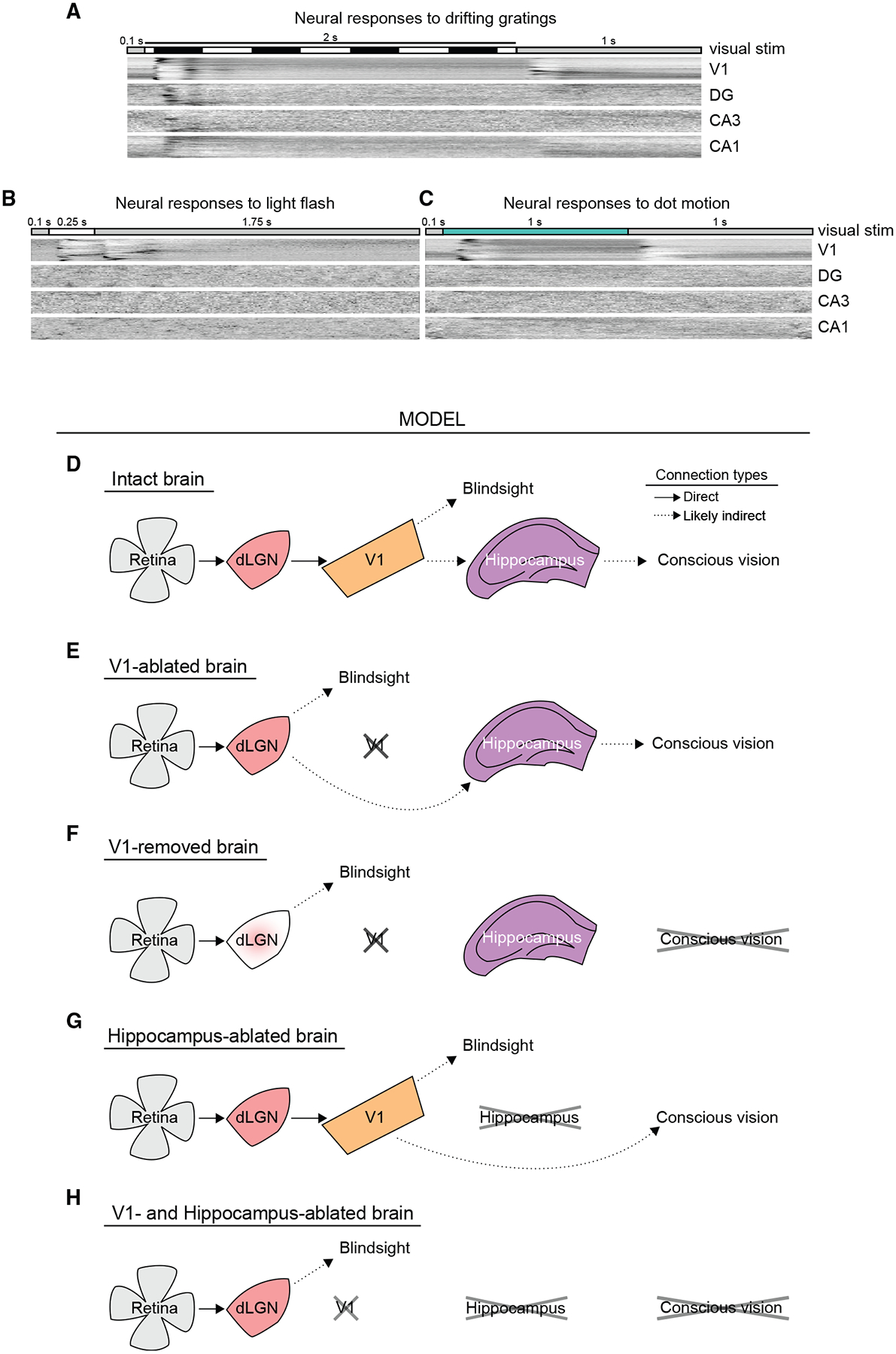
The hippocampus responds to drifting gratings and a model of the relationship between brain areas for putatively conscious vision and blindsight (A) Drifting gratings cause responses in subregions of the hippocampus (DG, CA3, and CA1) as well as V1. (B) A light flash does not cause a response in the hippocampus but does in V1. (C) Dot motion does not cause a response in the hippocampus but does in V1. (A–C) Each row is one neural unit sorted from multi-unit activity, with black indicating relative activation and white indicating relative suppression. (D) In the intact brain, results from muscimol suppression are consistent with a model in which dLGN, V1, and the hippocampus act serially for putatively conscious vision. (E) After unilateral ablation of V1 by ibotenate, there is little chronic effect on putatively conscious vision, consistent with compensation in which dLGN now functions upstream of the hippocampus, bypassing the loss of V1. (F) After unilateral removal of V1, putatively conscious vision is lost, suggesting that the neurons lost in dLGN support conscious vision via the hippocampus. The neurons that survive appear to support blindsight-like behavior. (G) After unilateral ablation of the hippocampus by ibotenate, there is no effect on vision, consistent with compensation in which V1 now bypasses the hippocampus for putatively conscious vision. (H) After simultaneous ipsilateral ablation of V1 and the hippocampus by ibotenate, putatively conscious vision is abolished because no additional brain area can compensate. Blindsight-like behavior persists due to the survival of the dLGN.

These results suggest that the hippocampus might respond to visual stimuli in the behavioral task. We analyzed Neuropixels electrophysiological recordings of the hippocampus available in the Allen Brain Observatory.^111^ Hippocampal neurons in the dentate gyrus (DG), cornu ammonis 3 (CA3), and cornu ammonis 1 (CA1) responded to large drifting gratings, which resemble the lateral visual targets that mice see while running in our task (Figure 7A). Hippocampal responses were specific to drifting gratings, as responses were weak, if at all present, to other stimuli that activated V1, such as light flashes (Figure 7B), dot motion (Figure 7C), dark flashes, static gratings, and natural scenes (data not shown).

Altogether, these results support the interpretation that V1 and the hippocampus function serially for conscious vision, but that in the permanently damaged brain, V1 and the hippocampus can each compensate for the loss of the other.

## DISCUSSION

We discovered pathways in the brain for putatively conscious vision using a new behavioral paradigm we developed for mice. Mice behave in a manner consistent with exhibiting both conscious and unconscious vision. Although determining with certainty that a mouse possesses conscious vision may be impossible, our finding that removal of V1 by suction causes behavior most consistent with a loss of conscious vision strongly justifies using this new model to identify functional pathways for putatively conscious vision.

We found that V1 and the hippocampus likely function in series in the intact brain to support conscious vision (Figure 7D). This conclusion rests on the striking effects of suppressing these areas with muscimol, in which silencing V1 abolishes all visual behavior toward contralateral targets, whereas silencing the hippocampus specifically blocks conscious vision. By contrast, selective ablation of V1 with ibotenate causes little blindness, suggesting that other brain circuits compensate, perhaps because the signal from dLGN now bypasses V1 to ultimately arrive at the hippocampus (Figure 7E). Such rapid compensatory mechanisms have been described in the cortex.^112^ We speculate that one pathway a signal could take from dLGN to the hippocampus is from dLGN to the higher visual areas,^113^ then to the perirhinal or postrhinal cortex, then to the entorhinal cortex, and finally into the hippocampus.^114^ Removal of V1 by suction compromises conscious vision, likely because it also causes extensive degeneration of dLGN, suggesting that conscious vision and its compensatory process require those neurons that die in dLGN (Figure 7F). Ablation of the hippocampus with ibotenate does not affect conscious vision during the chronic phase, suggesting that the brain compensates within this time interval by bypassing the hippocampus (Figure 7G). Dual ablation of V1 and the hippocampus with ibotenate specifically abolishes conscious vision during the chronic phase (Figure 7H).

We constructed a behavioral assay and completed salience reduction experiments to conclude that running to the central target on RC trials strongly suggested a loss of conscious vision. However, it remains possible that a reduction in salience under conditions not tested here might recapitulate the effect of V1 removal; therefore, further investigation is necessary to more fully rule out the salience interpretation. In this vein, new experiments that use the same stimuli to test for conscious vision and blindsight, rather than two different stimuli (RC and RO), will further clarify whether a loss of conscious vision is the best interpretation of these results.^115^

It is important to note that although we interpret the acute effects of suppression to reflect the normal function in the intact brain, it might be the case that suppression instead results in off-target effects.^57^ We also note that a caveat of muscimol injection is that its effective spread is difficult to precisely determine.

Bilateral ibotenate ablation of V1 caused a rat to lose visual discrimination ability.^80^ If a loss of visual discrimination is a good marker of a loss of conscious vision, this might put our studies in conflict, as unilateral ibotenate ablation of V1 caused a very small deficit in vision here. There are several technical differences between the studies, such as bilateral instead of unilateral ablations and unrestrained instead of head-fixed animals. Their sample size was also just one rat, leaving it unclear if their results generalize. If we ignore these caveats and compare our results at face value, one conclusion is that, to assess the presence of putatively conscious vision in an animal, a visual discrimination assay, despite being a good measure of one function of V1, is not a good substitute for training an animal to report whether they see a target. Another possibility is that the brain can compensate for a loss of conscious vision but cannot compensate for a loss of visual discrimination ability after V1 ablation.

Previous work used optogenetic methods to silence V1 in mice and showed deficits in visual behavior, though not the no sight that we see after injecting muscimol into V1.^74,76,81,82,84,85,87,88,116^ It is possible that the optogenetic methods used^117–119^ are not as effective as muscimol^120–122^ at silencing the visual cortex. Critically, our work should remind neuroscientists that not all lesion and inactivation methods are equivalent and that attention to the specific method may be necessary to assist in the interpretation of results.

If we draw an analogy between the results here with mice and the results from humans, the reason humans with lesions of V1 lose conscious vision might be because the LGN substantially degenerates, not because of V1 damage per se. Consistent with a role for LGN in conscious vision, LGN activity correlates with conscious visual perception in binocular rivalry.^123,124^ Second, the reason humans with hippocampal lesions do not lose conscious vision could be because pathways downstream of V1 compensate to bypass the hippocampus, as they appear to do in the mouse. Thus, there may be multiple routes in the human brain that are sufficient to support conscious vision that are activated only after damage to one of them. One prediction from these results is that suppressing the hippocampus in humans should cause an acute loss of contralateral conscious vision and perhaps a loss of all contralateral conscious perception, since signals from other senses also converge onto the hippocampus.

What function might the hippocampus play in conscious vision? It is well known that visual stimuli that appear after an initial stimulus can mask the visual experience of the initial stimulus.^13^ Visual masking demonstrates that the subjective experience of seeing is a process that results from analysis of a scene over time, sometimes referred to as the retentional model of perception.^125^ Since the hippocampus is critical for the very-short-term memory needed for trace conditioning (on the scale of hundreds of milliseconds),^126^ and since conscious vision integrates over a similarly short time window, we speculate that one role the hippocampus might play is as a very-short-term memory buffer specifically for the conscious aspect of vision. Suppressing the hippocampus causes this memory buffer to acutely dysfunction, and therefore a subject loses conscious vision.

Suppressing V1 results in no sight, including the absence of blindsight-like behavior. Since suppressing the hippocampus causes a mouse to lose conscious vision but retain blindsight-like behavior, this suggests that (1) blindsight can occur in the absence of compensation, and (2) blindsight normally depends on V1. Following permanent removal of V1, blindsight-like behavior exists, suggesting that the brain compensates such that unconscious vision becomes dependent on the surviving portion of dLGN.^25,29,127^ It remains possible that V1 does not play an instructive role in blindsight-like behavior but that its function is permissive, such that its suppression by muscimol reduces activation of brain areas in the blindsight circuit, such as SC, dLGN, or higher visual areas.^29,57^

This blindsight model supports the idea that animals and humans prioritize the use of conscious vision over unconscious vision.^128^ If an animal consciously sees a target in one position and unconsciously sees a target in a different position, they will act according to their conscious perception and ignore their unconscious perception, even if it will result in the wrong outcome (e.g., running to the unrewarded central target on RC trials). If an animal does not consciously see any targets but does unconsciously see a target, it will act based on its unconscious vision (e.g., running right on RO trials). Finally, if an animal neither consciously nor unconsciously sees a target, they will act largely as if they did not consciously see anything, meaning in a target-independent manner (e.g., running as if on blank trials).

Our work also informs the ongoing debate regarding theories of consciousness,^8^ such as global neuronal workspace theory (GNWT) and information integration theory (IIT). Neither of these theories postulates a central role for the hippocampus in conscious experience, yet this is what we find using our mouse model of blindsight. We expect that future work will leverage this model to test predictions made by various consciousness theories as well as to provide evidence for constructing a new, empirically driven theory, if needed.

In summary, we developed a mouse model of blindsight and used this model to reveal that the hippocampus plays a significant role in conscious vision. Mice possess the largest collection of tools available in any vertebrate animal for the manipulation of cell types, circuits, and brain areas. Using these tools, it should be possible to discover the neural circuits that function specifically for the conscious and unconscious components of visual perception. Comparing these two circuits should reveal fundamental principles for why some neural activities result in conscious experiences while others do not. Extending the blindsight model to other modalities in the mouse, such as touch,^129–131^ should further reveal the neural circuits that function for the conscious and unconscious components of touch perception, and comparing these circuits with those for vision should lead to the discovery of why the senses feel different. Altogether, such knowledge should facilitate the diagnosis of consciousness in other organisms as well as the assessment and perhaps inducement of consciousness in non-biological computational systems, if it turns out that consciousness is a product of specific abstract functional relations rather than specific physical processes.

## RESOURCE AVAILABILITY

### Lead contact

Requests for further information and resources should be directed to and will be fulfilled by the lead contact, Nikhil Bhatla (nikhil@iam.science).

### Materials availability

This study did not generate any new, unique reagents.

### Data and code availability

- All data are available from http://iam.science/data.
- All code is available from http://github.com/nbhatla/MiceVR/tree/KeepFromEscape.
- Any additional information required to reanalyze the data reported in this paper is available from the lead contact upon request.

## STAR★METHODS

### EXPERIMENTAL MODEL AND STUDY PARTICIPANT DETAILS

#### Mice

The Animal Care and Use Committee of the University of California, Berkeley approved all mice experiments. We purchased adult C57BL/6 wild-type female mice from Charles River for most experiments; in some cases, we bred wild-type females on site. In addition, we used adult female and male mice when testing transgenic mice. For suction experiments, the majority of mice were wild-type with some mice transgenic: Scnn1a-tg3-Cre; Ai9 (JAX 9613 crossed with 7909,^137^ which labels V1 and S1 clearly such that fluorescence was used to help localize suction in these mice), Pv-Cre; Ai32 (JAX 17320^138^ crossed with 24109^139^), Pv-Cre; Ai9 (JAX 17320 crossed with 7909), and Sst-Cre; Ai9 (JAX 13044^140^ crossed with 7909). For ibotenate experiments, most mice were wild-type with some mice transgenic or mutant: Ai9 (JAX 7909), Bax heterozygote (JAX 2994^141^), Pv-Cre; Ai9, and Sst-Cre; Ai9. For muscimol experiments, most mice were wild-type with some mice transgenic: Ai9 or Scnn1a-tg3-Cre; Ai9. We singly housed all trained mice, who weighed between 15–30g. None participated in any previous study. We maintained mice on a reverse light dark cycle, either 12 hours dark and 12 hours light or 14 hours dark and 10 hours light.

#### Humans

A previous study,^142^ approved by the University of Oxford Interdivisional Research Ethics Committee (IDREC; R59810/RE001 and R60132/RE001), acquired the MRI data that we analyzed here.

### METHOD DETAILS

#### Virtual reality apparatus

We assembled each virtual reality (VR) rig on a 2 ft by 2 ft steel board (Thorlabs MB2424) on which we clamped 3 computer monitors (Dell P2016 60 Hz or Asus PG258Q 240 Hz), a custom 3d-printed ball-holder (Fictiv), and a flexible lick-port holder (Fowler 52-585-015). We placed the central monitor in the mouse’s fronto-parallel plane at 25 cm, and we placed the lateral monitors at a 60° angle relative to the central monitor, such that the monitors encompassed the front 180° of the mouse’s visual field. The mouse ran on a foam ball (200 mm polystyrene, Graham Sweet Studios) which floated on an air stream provided by the building’s compressor. We surgically implanted custom stainless steel headplates on each mouse’s skull to facilitate head fixation. A headplate holder (Thorlabs C3A, modified by cutting with a Dremel) held the mouse in place above the ball via screws (2–56, 3/16” screws, McMaster 92196A076) through the headplate. An optical sensor (Logitech G403) placed in front of the ball detected the pitch and yaw rotations of the ball caused by the mouse. In the VR game, changes in pitch moved the mouse forward or backward while changes in yaw rotated the mouse. A computer (Raspberry Pi 3) running custom software sent ball movements over UDP to a second piece of custom software, called MiceVR, running in the Unity game engine (2017.3.0f3) on a Windows 10 computer (Intel i5-8600, 16GB RAM, Ge-force GTX 1060 3GB, Polywell). Gravity provided the pressure for dispensing water reward. A Windows computer running MiceVR opened a valve (12 VDC solenoid valve, American Science & Surplus) via an Arduino Uno to dispense water reward. 1/8” OD copper tubing (McMaster 8967K86) connected the valve to a reward port (18g blunt needle) placed in front of the mouse. Six of these rigs trained six mice simultaneously for 30–60 minutes per session, with up to 84 mice trained daily.

#### Eye tracking

We mounted two cameras (Basler Ace ACA1300–200UM) above each monitor and zoomed in (Computar MLH-10x zoom lens) to capture magnified images of both mouse eyes (Figure S4A). We mounted an on-axis infrared emitter (e.g. Phenas 48-LED IR illuminator) directly below each camera to (1) enable contrast so the pupil was visible, and (2) provide a reference corneal reflection (CR) that software used to calculate angular rotations of the eyes.^143^ Unity, via the Arduino, triggered the cameras to capture an image on each display frame of the video game (60 Hz). After recording eyes during a training session, custom Matlab software (Mathworks) automatically processed videos overnight. Briefly, to automatically identify the pupil, software binarized each video frame, removed high frequency occluders (e.g. flecks of polystyrene), inverted the image so that the pupil was white, found and filtered connected components (objects), excluded objects that were too small, and designated the pupil as the object that maximized a weighted sum of size, solidity and the inverse of the distance of the object from the previous frames’ pupil position. The software detected the CR as the brightest white object closest to the center of the video frame. The software automatically tracked pupil and CR positions with very high accuracy. Initially, we measured the radius from the center of rotation of the eye to the pupil (R_p_) for each eye for each mouse using a custom eye meter. In the eye meter, the mouse was head-fixed and we rotated a camera and IR emitter through a known angle (Thorlabs DTS25) while observing the displacement of the CR in the video. Since R_p_ varies as a function of pupil dilation,^144,145^ we varied the ambient illumination across multiple recording sweeps. We measured R_p_ as a function of pupil dilation for 129 mice across 11 strains. For subsequent mice, we stopped measuring R_p_ and instead used a conservative value of b = 0.925 with the previously found m = −0.142.^145^ Together, these parameters were more conservative than 96% of the observed data, meaning they overestimated eye movements. Overestimation was prudent in our case because we wanted to ensure that mice were not able to move a lateral target into the contralateral field, thereby cheating instead of using blindsight to correctly locate the target. With the CR position, pupil position and R_p_, the software calculated the horizontal change in pupil position in degrees^143^ throughout each video (Figure S4). The mean eye position during a single session was set to equal 0° in the azimuth plot. Since the software also knew where the lateral target was located on the screen on each video frame, the software calculated how often the target moved from its starting side to the opposite visual hemifield of the mouse after accounting for the mouse’s gaze position (Figure S4F). To be ready for surgery, a mouse must move the right lateral target into the left visual field, after accounting for eye movements and field restriction, on 20% or fewer of RC and RO trials.

#### Automatic watering system

To maintain water restriction over the weekends without requiring manual watering of the mice, we designed and built a custom automatic watering system. A custom printed circuit board functioned as a valve controller HAT on top of a Raspberry Pi computer to provide power management and connectivity to 4 custom valve driver boxes. We made two of these Pi HAT + computer devices. Communication was over I^2^C (PCA9615, MCP23017) using RJ45 8-wire connectors (CAT5 or CAT6). Each driver box controlled 16 valves (12 VDC solenoid valve, American Science & Surplus), which hung from multiple manifolds (such as Pneumadyne M20–250-10) which were each connected upstream to a 60 ml reservoir of water, allowing gravity to drive the flow of water when the valve opened. Rubber tubing connected each valve to a rigid copper pipe that inserted into each mouse cage via a flap grommet (Lab Products Super Mouse Microisolator AN074). The copper pipe connected to a custom stainless-steel reservoir (Affinity Manufacturing, Canada) that collected the water when the computer dispensed it. Copper pipe was critical because rubber tubing mysteriously filled with bubbles over the course of a week, which would occlude the tubing and restrict water delivery. We housed mice individually so that they did not compete for water delivered into their cage. We connected the system to the Internet so we could access it remotely and it could issue alerts. At a fixed time on each day of weekends and holidays (5 pm), the system would open each valve for a precise duration and then close the valve before opening the next one, where the open time defined how much water a mouse received. Custom software monitored each mouse’s weight as recorded on a Google Sheet during the prior week and then adjusted the amount of water dispensed to each cage depending on the amount of water needed over the prior weekend. In this way, each mouse received an individualized amount of water to maintain their weight over the weekend. The system worked reliably such that mice survived the weekend with their weight restricted, so they were ready for behavioral training on the next working day (e.g., Monday). This system supported up to 128 water-restricted cages, and we used approximately 90 cages to simultaneously water restrict 90 mice.

#### Behavioral training

##### Habituation and basic training

After implantation of the headplate, the mouse was in recovery until their weight reached the pre-surgery level, after which water restriction began. We restricted the amount of water that the mouse could consume over several days until their weight reached 80% or less of their starting weight, at which point training in VR began. Each VR rig trained mice each week day (5 days per week). Here we indicate the specific name for each level of training, as the custom software is provided in a public repository (see software section below). We tried 1,565 different levels before arriving at the standard training scheme described here. First, the VR rig trained mice to control the ball by initially placing mice in a virtual linear corridor (1_BG_ad0) at the end of which was a checkerboard target identical to the central target on the 3-choice task. Initially, the target was within a few steps of the mouse, and as the mouse successfully navigated to it the target adaptively moved farther away, so that the mouse had to maintain good ball control for a longer duration to reach the single target to collect reward. Once the mouse achieved criterion running on this level (4 trials/min), they advanced to the second level (1_BG), in which the target was at the end of the linear corridor in the same place it would be on the 3-choice task. Once the mouse reached criterion (4 trials/min over 3 consecutive days), the mouse advanced.

##### 3-choice training

The mouse began the 3-choice task without any field restriction and with only left-center (LC), center-only (CO) and right-center (RC) trials (3_BG_NoCo_AS). The checkerboard pattern painted on the cylindrical targets was set to a spatial frequency of 0.04 cycles per degree, the mean preferred spatial frequency for V1 neurons,^146^ when viewed from the mouse’s starting point on each trial. The VR rigs trained the first batch of V1-suctioned mice (11 mice) with a central target painted with horizontal stripes (3_BG_NoCo); the VR rigs trained all subsequent mice with a central target painted with a checkerboard pattern (3_BG_NoCo_AS). Correct trials ended with a black screen for two seconds before the start of the next trial, while incorrect trials ended with a static black-and-white screen for four seconds before the start of the next trial. To overcome any motor bias by the mouse, a bias correction algorithm automatically placed the correct target on the side away from the mouse’s turning bias.^147^ The algorithm selected the trial type for the next trial based on the mouse’s action history, calculating the probability of the target being in position X as

$$P(\text{target}\;\text{position}=X)=\frac{1-A_{X}}{\sum_{N}\left(1-A_{N}\right)},$$

where X is left, center or right, A_X_ is the fraction of actions in the mouse’s action history in which the mouse navigated to position X, and N is one of left, center and right. For example, if the mouse ran to the left 100% of the time (strong left bias), the next trial would never be an LC trial, and the algorithm would select with equal probability either a CO or RC trial. Once the mouse achieved criterion (>80% correct at each target location), we implemented nasal field restriction (Figure S4E), in which the nasal edge of the target would disappear if it crossed the line on the screen that was at a specified azimuth. The specified azimuths were −45°, −30°, −15°, −10°, −5°, 0°, 5°, 10°, 15°, and 20°. The mouse advanced to the next field restriction if it met criterion on the previous one for two consecutive days or simply one day if it was the first day at that field restriction. If after reaching a field restriction of 20° the mouse could still move right targets into their left visual field with eye movements on more than 20% of right target trials, we advanced field restriction to 25° and again to a maximum of 30° as needed. Once the mouse performed well with enough field restriction and reached behavioral criterion, we disabled bias correction, the software selected each trial randomly (i.e. each trial type picked on 1/3 of trials) and enabled correction trials, in which errors made on LC, CO or RC trials caused the same trial to be repeated until mouse made the correct action (3_BG_AS). We excluded performance on correction trials from all analyses that we presented. Once the mouse reached criterion, the next level (3_BG_Bl_AS) was one in which the software selected trials from blocks rather than randomly to ensure more equal presentation of each trial type. Once the mouse reached criterion, on the next level (3_BG_Bl_R_10_AS) we reduced RC trials to 10% of trials while increasing CO and LC trials to 45% each. We did this in anticipation of the mouse exhibiting blindness following surgery and not wanting to encourage the mouse to use blindsight if they experienced a high frequency of errors on RC trials; researchers used a similar strategy with monkeys.^62^ Once the mouse reached criterion, on the final level (3_BG_Bl_R_10_Ca_2-p5_Ext_2p5_AS or 3_BG_Bl_R_10_Ca_7p5_Ext_7p5_AS) we introduced left-only (LO), right-only (RO) and blank trials on 2.5% or 7.5% of trials each, reducing RC trials to 7.5% and LC and CO trials to 42.5% or 35% each, respectively. Moreover, corrections on this level only occurred for LC, LO, and CO trials; as above, if a mouse is blind on the right side, we did not want to give them many trials to learn how to correct their errors. The mouse must perform above criterion (>80% accurate on each trial type) on average over at least five consecutive days before deemed ready for surgery. Mean training time to mastery of the final level before surgery, including field restriction, for the mice presented here was 54 training days (range, 29 to 136, n=107 mice). 30% of head-plated mice failed to advance to post-surgery because (a) the headplate fell off during training prior to surgery, (b) the mouse failed to learn despite multiple resets back to the starting level, or (c) an unexpected demise. During this study, we attempted to train a total of 153 mice. Successfully trained mice had an average age at headplate surgery of 102 days (range, 49 to 192) and an average age at neural manipulation surgery of 240 days (range, 119 to 526).

##### Salience experiments

We trained mice in the standard 3-choice task, and then we reduced the opacity of the right target until each mouse ran to the central target on RC trials. For two mice, we first reduced the opacity from 100% to 50% to 6% to 3% (3_BG_Bl_R_15_Ca_7p5_Ext_7-p5_R_Opa_XX_AS), and found that 3% opacity produced reliable running to the central target on RC trials whereas 6% did not. For the remaining five mice, 6% opacity was sufficient to induce running to the central target on RC trials, with mice either progressively approaching 6% opacity (1 mouse) or immediately jumping from 100% to 6% opacity (4 mice) (Figure 2H). One mouse was lost after low salience testing but prior to V1 removal, reducing the total number of mice from seven to six.

##### 4-choice training

Once the mouse mastered the 3-choice task, we trained some on the 4-choice task. On the first level (4_BG_NoCo), the mouse must run to the location of the visual target at one of four possible locations. Once the mouse reached criterion, we increased field restriction as with 3-choice training. We then enabled corrections (4_BG), then blocks (4_BG_Bl), and then corrections only for left trials (4_BG_Bl_L_Co). Once the mouse reached criterion, we advanced them to 4_BG_Bl_R_10_Ca_2p5, where software presents the near-right and far-right targets on 5% of trials each and blank trials occurred 2.5% of the time. Correction trials did not occur after right trials. If the mouse reached criterion, then we presented the mouse with the final 3-choice level again (3_BG_Bl_R_10_Ca_2-p5_Ext_2p5) on the following day, and if the mouse reached criterion, we alternated the mouse between 4-choice and 3-choice tasks for at least five cycles (10 days), at which point the mouse was ready for surgery. One mouse failed to master the standard X-shaped version of the 4-choice task (Figure 3B) and instead mastered the alternative F-shaped version (Figure 3F). We pooled the results from both variants of the 4-choice task in Figure 3.

#### Neurosurgery

##### Suction removal of cortex

During headplate surgery, we recorded a photograph of the exposed skull showing the suture lines because during the training period the exposed suture lines disappeared. Using this photograph as a guide, we made a craniotomy of approximately 3 mm in diameter above left V1 using a 400 um drill bit in a dental drill (Foredom MH-170). The craniotomy extended laterally along the anterior edge of the lambdoid suture line, from 1.5 mm to 4.5 mm to the left of the midline and 3 mm anterior of the lambdoid suture line. After removing the skull flap, we removed the dura and used a 20 g blunt needle (CMLsupply) connected to the building’s vacuum for the first pass of aspiration. We used Scnn1a-Cre; Ai9^137^ mice in some cases (8 of 35 mice), in which fluorescence selective to V1 guided aspiration. We used Pv-Cre; Ai9 mice in some cases (4 of 35 mice). We used an Sst-Cre; Ai9 mouse in one case. The remaining 22 mice were wild-type. Aspiration proceeded until white matter was visible. In many cases the white matter remained, but in some cases, aspiration proceeded further until the grey matter of the hippocampus was visible, at which point we stopped aspiration. Using a 21 g needle, we took care to aspirate along the caudal edge of the craniotomy by lowering the aspirator below the posterior bone, focally targeting the lateral caudal pole of V1. Working in this way occasionally exposed the cerebellum and colliculi without damaging them. In some cases, a significant amount of bleeding occurred so we used a cauterizer (Fine Science Tools 18000–00) to suppress bleeding. We glued a 4 mm circular cover slip to cover the craniotomy after aspiration was complete. For aspiration of primary somatosensory cortex (S1), we made a craniotomy from 2.5 mm to 5 mm to the left of the midline with the caudal edge 2 mm anterior of the lambdoid suture line and the rostral edge just caudal to the bregma suture line. We tested mice on behavior either the next day or 10 days later, for at least 5 days and at most 31 days. We trained and tested eight mice without field restriction, and we trained and tested the remaining 27 mice with field restriction. We tested 28 mice the next day after aspiration, and we tested 7 mice after 10 days. We trained and tested two mice with solid black targets, one mouse with solid white targets, and 32 mice with black-and-white targets. Of the 32 mice, we trained 12 with the central target having only horizontal bars and 20 with the central target matching the lateral targets with horizontal and vertical bars (checkerboard), as shown in Figure 1. We used post-mortem histological analysis (see histological analysis section below) to exclude mice from behavioral analysis if less than 80% of left V1 was removed (4 mice excluded).

##### Muscimol suppression

We trained mice on a special 3-choice level (3_BG_Bl_R_15_Ca_7p5_Ext_7p5_NoCo_AS) prior to injection, in which we disabled correction trials and blank, RO and LO trials were increased to 7.5% each, with RC trials at 7.5% and LC and CO trials at 35% each. We increased the number of blank, RO and LO trials because we ran mice for only one session following muscimol injections and then perfused immediately. Mice were injected (World Precision Instruments UMP3 UltraMicroPump driven by a Micro4) with 2.25 mM muscimol-BODIPY (Thermo Fisher M23400 dissolved in 1x phosphate-buffered saline) into the following brain areas with the following parameters: V1, either three injection sites (−2 mm from midline, lambda; −3.5, lambda; −2.75, lambda + 1; depth = 250 um, volume = 200 nl, flow rate = 25 nl/min, n = 3 mice) or one injection site (−2.75, lambda + 0.5; depth = 250, volume = 300, flow rate = 25, n = 2 mice); dLGN, −2, −1.75 to −2.25 from bregma, depth = 2150–2200, volume = 100–200, flow rate = 100–300; VP, −2.5, −1.75 from bregma, depth = 2500, volume = 100, flow rate = 100; and hippocampus, −2, −2.25 from bregma, depth = 1300–1800, volume = 100–200, flow rate = 100–300. Behavioral testing resumed at least 2 hours following injection on the same level the mouse was trained on the previous day. Mice were perfused immediately following behavioral testing, and histology proceeded without NeuN antibody staining. We confirmed injection sites by aligning the slices to the Allen Brain Atlas (see histological analysis section) and observing the extent of fluorescence. We localized fluorescence to each brain region and we excluded mice in which fluorescence was outside of a targeted brain region.

##### Ibotenate ablation

1% ibotenic acid (Abcam ab120041 dissolved in 1x phosphate-buffered saline) was injected into the following brain areas with the following parameters: the hippocampus alone, three injection sites (−2 mm, lambda; −3.5 mm, lambda; −2.75 mm, lambda+1), depth = 1500 um, volume = 100 nl, flow rate = 50 nl/min; V1 alone, three injection sites (−2, lambda; −3.5, lambda; −2.75, lambda+1), depths = 400 and 150, volume = 175–250 nl/depth, flow rate = 25–100; V1 + hippocampus, three injection sites (−2, lambda; −3.5, lambda; −2.75, lambda), depths = 400 and 150um, volume = 250–500/depth, flow rate = 50–100; superior colliculus partial ablation, one injection site (−0.5, lambda), depths = 1500, 1400 and 1300um, volume = 200/depth, flow rate = 20; superior colliculus total ablation, two injection sites (−0.5, lambda; −1.5, lambda-0.5), depths = 1500, 1350 and 1200 um, volume = 200/depth, flow rate = 20. We gave mice either 4–8 hours to recover or 10 days to recover, and then tested for 20 days followed by perfusion. We excluded the behavioral performance observed on the same day as ibotenate injection for both V1 and hippocampus injections, because the mice had a strong ipsiversive bias (left action on all trials: pre-ibotenate = 46%, post-ibotenate = 84%, p = 0.01; n = 4 mice for V1 injection). The acute behavioral effects of muscimol and ibotenate are likely different because muscimol hyperpolarizes neurons whereas ibotenate causes pathological depolarization. We quantified the extent of ablation as described in the histological analysis section below.

#### Histology

We perfused mice intracardially with 4% paraformaldehyde (PFA) and then removed the brain using either a dorsal or ventral approach; the ventral approach reduced damage occasionally caused by removing the headplate. We kept brains in PFA overnight at 4° C and transferred to 30% sucrose solution at room temp. for one or two days until the brain sank. We cut 40 um sections on a freezing microtome (American Optical 860), keeping every third or fourth section. For the anti-NeuN stain, we incubated slices in blocking solution containing 1:10,000 dilution of rabbit anti-NeuN antibody (Abcam ab177487) overnight at 4° C. We then washed slices in PBS-T, and then incubated them in blocking solution containing 1:500 or 1:1,000 dilution of anti-rabbit secondary antibody (Alexa Fluor 405, 488 or 555, Thermo Fisher) for at least 1 hour at room temperature. Then we washed slices in PBS-T and mounted them on slides and coverslipped (Vectashield+DAPI Hardset, H-1500). We recorded images on a macroscope (Olympus MVX10, 0.63x and 2x objectives) at varying zoom magnifications. For AChE staining, we followed the protocol from McDonald et al.^148^ Briefly, we incubated each 40 um slice at room temperature or 4 °C overnight in 500 ml of double-distilled water containing 500 mg acetylthiocholine iodide, 375 mg glycine, 250 mg cupric sulfate, and 2140 mg anhydrous sodium acetate, titrated to pH 5.2 with acetic acid. We added ethopropazine (1 mM) to inhibit non-AChE cholinesterases. We visualized the reaction product using 2% potassium ferri-cyanide in distilled water at room temperature for 5–10 min. We rinsed sections in 3 changes of 0.9% saline and then mounted and imaged them with white light. We located V1 based on induced darkening in layer 4 of cortex (Figure S2). We measured dLGN degeneration after mice completed post-surgery behavioral testing. For ibotenate, we defined dorsal hippocampus as the portion of hippocampus that was dorsal to the peak of the lateral bulge of the medial geniculate when viewed in coronal sections. More precisely, the dorsal hippocampus’ rostral boundary was −0.95 mm bregma and caudal boundary was −3.79 mm bregma, dorsal boundary as high as 1.5 mm deep and ventral boundary as low as 3.5 mm deep, depending on the position along the rostral-caudal axis. We included all subdivisions of the hippocampus included in these boundaries as the dorsal hippocampus. For muscimol suppression, we observed the focus of muscimol injection to be at the dorsal dentate gyrus located at approximately −2.7 mm bregma, 2.3 mm deep and 2.2 mm lateral from the midline.

### QUANTIFICATION AND STATISTICAL ANALYSIS

#### Histological analysis

##### Brain area identification

First, we localized V1 in coronal serial sections using acetylcholinesterase (AChE) staining^149^ in a subset of mice. We then confirmed that this location corresponded to the location of V1 annotated when we semi-automatically aligned brain sections to the Allen Mouse Brain Atlas^132,134^ using the Fiji plugin Aligning Big Brains and Atlases (ABBA,^132^ https://biop.github.io/ijp-imagetoatlas/). We manually corrected alignments in QuPath.^133^ These corrections started by ensuring that the midline was accurate by placing the region-of-interest (ROI) for retrosplenial cortex (RSP) so that it bordered the midline of the brain. Visual cortex is directly medial to RSP, and we shifted ROIs within the visual cortex laterally to ensure no overlap and no gaps with the RSP ROI. We then adjusted ROIs dorsoventrally to ensure inclusion of layer 1 of cortex as well as exclusion of the underlying white matter. Localization of V1 by AChE staining was like that found by Allen Atlas registration (Figure S2). For all remaining experiments, we then relied on the Atlas annotations of all brain areas of interest and quantified the extent of removal of each brain area for each mouse. We used custom scripts in QuPath for quantification.

##### Quantification of brain area removal

To quantify the size of removal of left visual areas, including V1, we duplicated the ROIs generated by ABBA and then adjusted these duplicates to match the boundaries of the amount of brain remaining. Then, we calculated the fraction of left V1 removed by

$$\text{Fraction}\;\text{of}\;\text{left}\;\text{V}1\;\text{removed}=1-\frac{\sum_{\text{slices}}{\text{Actual}\;\text{Area}}_{\text{left}\;V1}}{\sum_{\text{slices}}{\text{Expected}\;\text{Area}}_{\text{left}\;V1}}.$$

We excluded mice in which we removed less than 80% of V1 from Figures 1E and 1K. We also measured the other visual cortical areas and hippocampal CA1 in this way (Figure S1).

##### Quantification of dLGN neuron degeneration

To quantify degeneration of neurons in dLGN, we used QuPath’s cell detection feature to automatically annotate neurons labeled in dLGN in high-magnification anti-NeuN-stained slices. We manually tuned cell detection parameters for each slice such that automatic cell detection was highly accurate. Then, we calculated the fraction of left dLGN that degenerated by

$$\text{Fraction}\;\text{of}\;\text{left}\;\text{dLGN}\;\text{killed}=1-\frac{\sum_{\text{slices}}{\text{Neuron}\;\text{count}}_{\text{left}\;\text{dLGN}}}{\sum_{\text{slices}}{\text{Neuron}\;\text{count}}_{\text{right}\;\text{dLGN}}}.$$

Second, we calculated the dimming of neurons in dLGN by

$$\text{Left}\;\text{dLGN}\;\text{neuron}\;\text{dimming}=1-\frac{{\text{Mean}\;\text{neuron}\;\text{brightness}}_{\text{left}\;\text{dLGN}}-{\text{Mean}\;\text{background}\;\text{brightness}}_{L}}{{\text{Mean}\;\text{neuron}\;\text{brightness}}_{\text{right}\;\text{dLGN}}-{\text{Mean}\;\text{background}\;\text{brightness}}_{R}}.$$

Finally, we calculated shrinkage of neurons in dLGN by

$$\text{Left}\;\text{dLGN}\;\text{cell}\;\text{shrinkage}=1-\frac{{\text{Mean}\;\text{neuron}\;\text{area}}_{\text{left}\;\text{dLGN}}}{{\text{Mean}\;\text{neuron}\;\text{area}}_{\text{right}\;\text{dLGN}}}.$$

We measured dLGN degeneration after mice completed post-surgical behavioral testing and were perfused. For Figure 1, some of the V1-removed mice (17 of 30) underwent additional surgeries after the testing period that affected the right dLGN. For those mice, instead of comparing dLGN neuron statistics between the left and right sides in the same mouse, we compared the left dLGN with the statistics of the average right dLGN, which we calculated from 13 mice that did not undergo any additional surgeries. For statistics presented on the V1-removed mice in Figure 4, we only analyzed these 13 mice. Within these 13 mice, there were two cohorts, with the first cohort perfused and examined 5–9 months after surgery and the second cohort perfused and examined 6–7 weeks after surgery. We did not see a significant difference between these two batches, so we pooled those results. For V1 ibotenate, mice were perfused 4–7 weeks after surgery. While being perfused slightly sooner, we do not believe that this affected our results, for the following reasons. First, nearly all of the dLGN degeneration that occurs after V1 aspiration occurs within 2 weeks of surgery.^92^ Second, if we compare only brains that were perfused 6–7 weeks after surgery for both aspiration and ibotenate, all the conclusions from Figure 4 continue to hold.

##### Quantification of brain area ablation by ibotenate

To quantify death in V1 by ibotenate, we used QuPath’s cell detection feature to automatically annotate neurons labeled in V1 in high-magnification anti-NeuN-stained slices. We manually tuned cell detection parameters for each slice such that automatic cell detection was highly accurate. Then, we calculated the fraction of left V1 that we ablated by

$$\text{Left}\;V1\;\text{death}=1-\frac{\sum_{\text{slices}}{\text{Neuron}\;\text{count}}_{\text{left}\;V1}}{\sum_{\text{slices}}{\text{Neuron}\;\text{count}}_{\text{right}\;V1}}.$$

To quantify death by ibotenate in the hippocampus,

$$\text{Left}\;\text{Hippocampus}\;\text{death}=1-\frac{\sum_{\text{slices}}w_{L}\left({\text{Mean}\;\text{brightness}}_{\text{Left}\;\text{Hip}}-{\text{Mean}\;\text{background}\;\text{brightness}}_{L}\right)}{\sum_{\text{slices}}w_{R}\left({\text{Mean}\;\text{brightness}}_{\text{Right}\;\text{Hip}}-{\text{Mean}\;\text{background}\;\text{brightness}}_{R}\right)}$$

where L = left, R = right, and the weights w are the fraction of the area for the brain area on that slice divided by the total area of the brain area across all slices. We found the mean background brightness by specifying a small region of interest that was not fluorescing near the brain area for each slice.

#### Measures of behavior

We summarized the behavioral response to neurosurgical manipulations using two measures:

$$RC\;\text{adjusted}\;\text{accuracy}=\frac{R_{RC}-R_{CO}}{1-R_{CO}}.$$

$$RO\;\text{adjusted}\;\text{accuracy}=\frac{R_{RO}-R_{B}}{1-R_{B}}.$$

where R_RC_ is the fraction of right actions on RC trials, R_CO_ is the fraction of right actions on CO trials, R_RO_ is the fraction of right actions on RO trials, and R_B_ is the fraction of right actions on blank trials. On 4-choice,

$$4R\;\text{adjusted}\;\text{accuracy}=\frac{\left(FR_{FR}+NR_{NR}\right)-\left(FR_{B}+NR_{B}\right)}{2-\left(FR_{B}+NR_{B}\right)},$$

where FR_FR_ is the fraction of far-right actions on FR trials, NR_NR_ is the fraction of near-right actions on NR trials, FR_B_ is the fractions of far-right actions on blank trials, and NR_B_ is the fraction of near-right actions on blank trials. We calculated a trial’s response latency as the amount of time the mouse took from when it could move in a trial (two seconds after trial start) to when it ran to one of the available target locations. We averaged the response latencies for a specific action on a specific trial type for a specific mouse, and then averaged these averages to get the mean response latency across a cohort of mice.

#### Running trajectory analysis

To plot the average running trajectory for a mouse for a specific trial type, software down sampled the running trajectory for each trial of that type to the minimum trial length of that set of trials, such that all trajectories were the same length. Software calculated and plotted the average position for each time point. The thickness of the trajectory line shows the 95% confidence interval for the distribution of positions at that timepoint. We did not depict the confidence interval in the forward/backward direction because that is in the direction of motion and so is more difficult to visualize;the confidence interval in the left/right direction is clearly indicated by the thickness of the mean trajectory line (Figure S5).

#### Magnetic resonance volume analysis

We used a combination of tools from the FMRIB Software Library (FSL) to process, view, and annotate MR images.^135^ We used the Brain Extraction Tool (BET) to isolate the brain, then registered the isolated image to the MN152_T1 1mm standard using FMRIB’s Linear Registration Tool (FLIRT). We viewed the registered images in FSLeyes, which displayed the sagittal, coronal, and horizontal planes side by side. We determined the general location and shape of the LGN using the Juelich Histological Atlas and annotated the left and right regions through a self-refining process, creating the annotations in the sagittal view, then revising the images in the coronal and horizontal views. We used a series of FSLmaths commands to determine the voxel counts of the resulting files, then took the ratios of the left and right. The commands were: fslmaths -mas to create a mask of the LGN annotation with reference to the original image; fslmaths -thr and -uthr to establish lower and upper brightness thresholds to use when counting voxels (generally -thr 500 -uthr 700, occasionally adjusted to account for image brightness); and fslstat -v to quantify final voxel count.

#### Allen Brain Observatory Neuropixels analysis

We analyzed data following https://allensdk.readthedocs.io/en/latest/visual_coding_neuropixels.html.^111^ Briefly, we used the provided Jupyter notebooks to download the extracellular electrophysiology data, extract units recorded in V1 (VisP) and hippocampus (DG, CA3 and CA1) during specific stimuli, and visualize the peristimulus histograms using Rasterplot^136^ with default parameters (automatic neuron binning, n_clusters=100, n_PCs=200). We analyzed the flashes (bright or dark, separately), natural_scenes, static_gratings, and drifting_gratings stimuli from the Brain Observatory 1.1 dataset and dot_motion stimuli from the Functional Connectivity dataset. We did not separate by mouse genotype or stimulus features, instead pooling across mouse genotype and stimulus orientation, contrast, temporal frequency, spatial frequency, phase, direction, and speed, where applicable for each stimulus type. For each stimulus type, we pooled all trials of that stimulus type and averaged the spiking activity of the provided sorted units. This means that for the natural_scenes stimulus type, since we did not group neural activity by individual images but pooled across all images, the neural response presented was a generic “change response” rather than the response to the onset or offset of an individual natural scene. The Jupyter notebook is available as described below in the software section.

#### Statistical analysis

Unless otherwise specified, we calculated all p values using two-tailed paired t-tests to compare changes within mice between trial types or pre- and post-surgery. To determine whether individual mice possessed blindsight-like behavior, we calculated the RC- and RO-adjusted accuracies (and 4R-adjusted accuracy, if available) for each day for at least 5 to 31 days of training. We then compared these distributions of RC- and RO-adjusted accuracies (and 4R-adjusted accuracy, if available) using a one-tailed paired t-test, since we expected RO adjusted accuracy to be larger than RC adjusted accuracy. We did not correct for multiple comparisons. For comparing the histological effects on dLGN of V1 suction with V1 ibotenate, we calculated p values using unpaired t-tests. *p < 0.05, **p < 0.01, ***p < 0.001; ns, not significant. r is Pearson’s r.

### ADDITIONAL RESOURCES

#### Software

All software and analysis code is available at https://github.com/nbhatla/MiceVR/tree/KeepFromEscape.

## Supplementary Material

1

2

Supplemental information can be found online at https://doi.org/10.1016/j.cub.2026.03.031.

## Figures and Tables

**Table:** KEY RESOURCES TABLE

REAGENT or RESOURCE	SOURCE	IDENTIFIER
Antibodies		
Rabbit Monoclonal Anti-NeuN antibody [EPR12763] - Neuronal Marker	Abcam	ab177487; RRID: AB_2532109
Goat anti-Rabbit IgG (H+L) Cross-Adsorbed Secondary Antibody, Alexa Fluor 405 - A-31556	Thermo Fisher	A-31556; RRID: AB_221605
Goat anti-Rabbit IgG (H+L) Cross-Adsorbed Secondary Antibody, Alexa Fluor 488 - A-11008	Thermo Fisher	A-11008; RRID: AB_143165
Donkey anti-Rabbit IgG (H+L) Highly Cross-Adsorbed Secondary Antibody, Alexa Fluor 555 - A-31572	Thermo Fisher	A-31572; RRID: AB_162543
Chemicals, peptides, and recombinant proteins		
Ibotenic acid, excitotoxic agonist	Abcam	ab120041
Muscimol, BODIPY TMR-X Conjugate	Invitrogen	M23400
Experimental models: Organisms/strains		
Mouse: C57BL/6	Charles River	#027; RRID: IMSR_CRL:027
Mouse: Scnn1a-Tg3-Cre	JAX	#9613
Mouse: Ai9	JAX	#7909
Mouse: B6 Pvalb-IRES-Cre	JAX	#17320
Mouse: Ai32	JAX	#24109
Mouse: Bax	JAX	#2994
Mouse: Sst-IRES-Cre mice	JAX	#13044
Software and algorithms		
MiceVR - mouse virtual reality system in Unity	This work	https://github.com/nbhatla/MiceVR/tree/KeepFromEscape
Aligning Big Brains and Atlases	Chiaruttini et al.^132^	https://biop.github.io/ijp-imagetoatlas/
QuPath	Bankhead et al.^133^	https://qupath.github.io/
Allen Reference Atlas – Mouse Brain	Allen et al.^134^	https://atlas.brain-map.org/atlas?atlas=1
FMRIB Software Library (FSL)	Smith et al.^135^	https://doi.org/10.1016/j.neuroimage.2004.07.051
Rastermap	Stringer et al.^136^	https://doi.org/10.1038/s41593-024-01783-4
Other		
Data for Figures 1, 2, 3, 4, 5, and 6 and Supplemental Figures	This work	http://iam.science/data
Data for Figures 7A–7C	Siegle et al.^111^	https://allensdk.readthedocs.io/en/latest/visual_coding_neuropixels.html

## Highlights

- The hippocampus plays a role specific to conscious vision in the intact brain
- V1 does not play a role specific to conscious vision in the intact brain
- V1, dLGN, and the hippocampus specifically support conscious vision in the damaged brain
- Suppression, ablation, and removal of a brain area can cause strikingly different effects
