# Acute requirement for the hippocampus in putatively conscious vision revealed by a mouse model of blindsight

**DOI:** 10.1016/j.cub.2026.05.048

**Published:** 2026-06-22

**Authors:** Nikhil Bhatla, Kathryn Cheong, Sho Takahashi, Corliss Lay, Matthew Fiedeldey, Kathleen Liang, Mo Mao, Yun-Fan Wu, Sandy Lu, Karis Yun, Viveca Ganti, Raymond Tsao, Xiaozhou Zhang, Kenzy A. Mohamed, Yul Chang, Yucong Geng, Casey Dai, Ashley K. Liu, Yee May Lwin, Heidi Kim, Vamsi Subraveti, Narumi Mitchell, Na Youn Jo, Umais Khan, Roger Anguera-Singla, Zephyr Weinreich, Hanna E. Willis, Holly Bridge, Michael P. Stryker, Hillel Adesnik

Following publication, a reader pointed out that the legends for Figures 1, 2, 3, 5, 6, and S6 stated "*p <0.5," when in fact it should be "*p < 0.05." They also noted that the legend for Figure 4 stated that we used a paired *t* test, when in fact we used an unpaired *t* test.

We apologize for any confusion caused by these issues. These errors have now been corrected and do not affect any of the conclusions of the paper.

