# Supplementary material for: Acute requirement for the hippocampus in putatively conscious vision revealed by a mouse model of blindsight
Source: Curr Biol. Author manuscript; Available in PMC 2026 Jul 23. (PMC13393572; doi:10.1016/j.cub.2026.03.031)
Supplement: 1 [file NIHMS2163924-supplement-1.pdf]

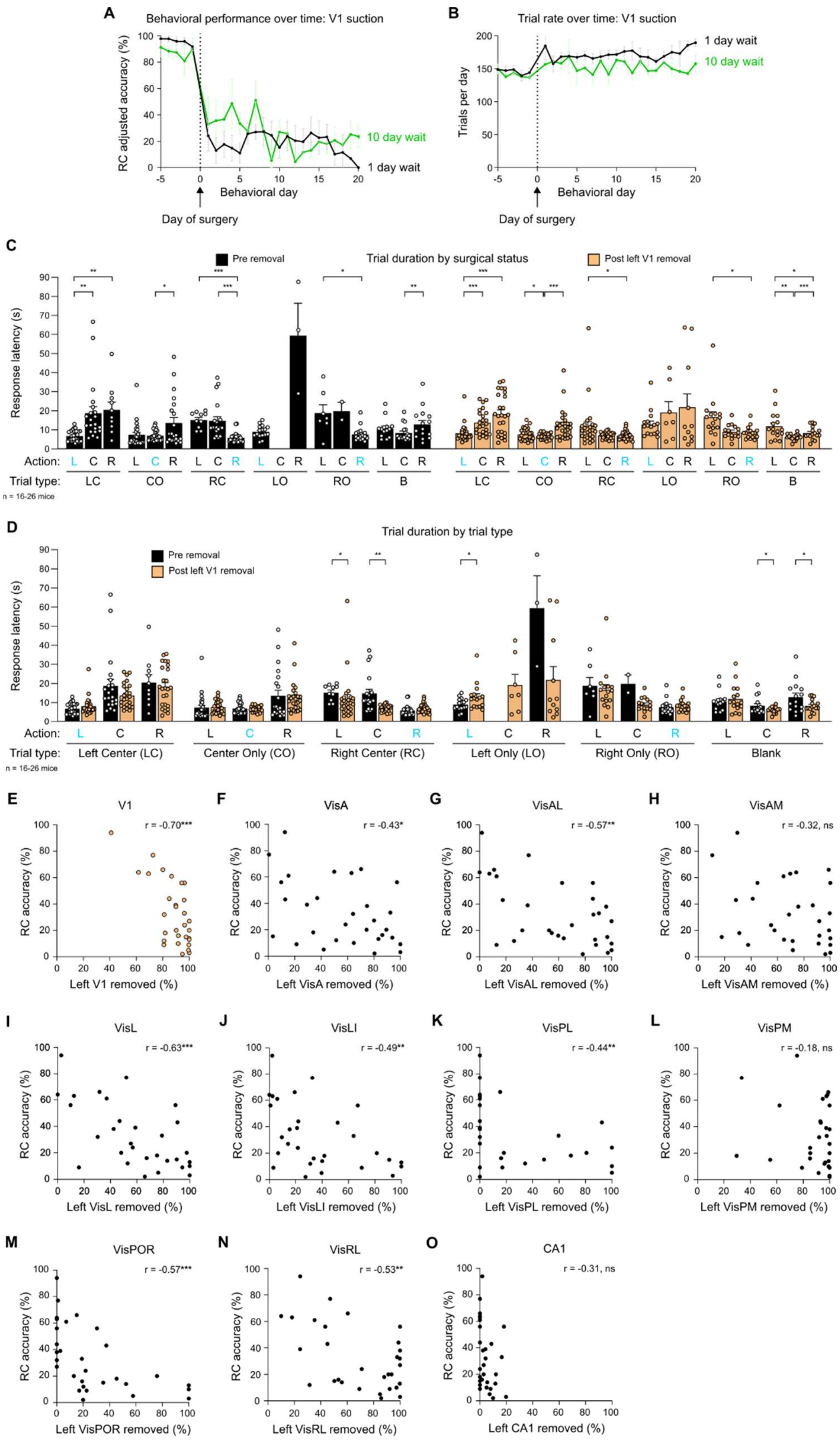

A
Behavioral performance over time: V1 suction
RC adjusted accuracy (%)
10 day wait
1 day wait
Behavioral day
Day of surgery
B
Trial rate over time: V1 suction
Trials per day
1 day wait
10 day wait
Behavioral day
Day of surgery
C
Trial duration by surgical status
Pre removal
Post left V1 removal
Response latency (s)
Action:
Trial type:
LC
CO
RC
LO
RO
B
n = 16-26 mice
D
Trial duration by trial type
Pre removal
Post left V1 removal
Response latency (s)
Action:
Trial type:
Left Center (LC)
Center Only (CO)
Right Center (RC)
Left Only (LO)
Right Only (RO)
Blank
n = 16-26 mice
E
V1
r = -0.70***
RC accuracy (%)
Left V1 removed (%)
F
VisA
r = -0.43*
Left VisA removed (%)
G
VisAL
r = -0.57**
Left VisAL removed (%)
H
VisAM
r = -0.32, ns
Left VisAM removed (%)
I
VisL
r = -0.63***
Left VisL removed (%)
J
VisLI
r = -0.49**
Left VisLI removed (%)
K
VisPL
r = -0.44**
Left VisPL removed (%)
L
VisPM
r = -0.18, ns
Left VisPM removed (%)
M
VisPOR
r = -0.57***
Left VisPOR removed (%)
N
VisRL
r = -0.53**
Left VisRL removed (%)
O
CA1
r = -0.31, ns
Left CA1 removed (%)

Figure S1. Varying the break duration after V1 removal does not affect behavioral performance, and V1 removal correlates best with blindness compared to removal of adjacent brain areas. Related to Figure 1.

(A) Surgery occurred on day 0, and behavioral day 1 corresponds to the first day of testing following either a 1-day wait or a 10-day wait. Mice were tested for up to 20 days following surgery. After V1 suction, RC adjusted accuracy does not recover with additional training and is largely equal regardless of the post-surgery break duration.

(B) Mice perform the same number of trials regardless of break duration following surgery.

(A-B) 1-day wait: n = 10 mice; 10-day wait: n = 6 mice.

(C) Amount of time to complete each action for each trial type, grouped by surgery status, then trial type, then action. Blue font indicates the rewarded action (receiving water). Rewarded actions generally have lower latencies than unrewarded actions.

(D) Identical data as (C), but grouped by trial type, then action, then surgery status.

(E) The more V1 is removed, the more blind the mouse is ($r = -0.70$***). Reproduced from Figure 1H.

(F) The more VisA is removed, the more blind the mouse is ($r = -0.43$*).

(G) The more VisAL is removed, the more blind the mouse is ($r = -0.57$**).

(H) VisAM removal does not correlate with blindness ($r = -0.32$, ns).

(I) The more VisL is removed, the more blind the mouse is ($r = -0.63$***).

(J) The more VisLI is removed, the more blind the mouse is ($r = -0.49$**).

(K) The more VisPL is removed, the more blind the mouse is ($r = -0.44$**).

(L) VisPM removal does not correlate with blindness ($r = -0.18$, ns).

(M) The more VisPOR is removed, the more blind the mouse is ($r = -0.57$***).

(N) The more VisRL is removed, the more blind the mouse is ($r = -0.53$**).

(O) Hippocampal CA1 removal does not correlate with blindness ($r = -0.31$, ns).

(E-O) Note that V1 was targeted and removed in all of these surgeries, so these results should not be interpreted to indicate that selective removal of a single non-V1 visual cortical area would result in blindness in the absence of V1 removal.

Each dot is one mouse. Error bars indicate SEM. * $p < 0.05$, ** $p < 0.01$, *** $p < 0.001$, ns is $p \geq 0.05$, paired t-test (C-D; unmarked pairs were found to be ns) or slope of linear regression (E-O). n = 30 mice for all plots except n = 16 mice for (A-B) and VisPL (K), and n = 16-26 mice for (C-D).

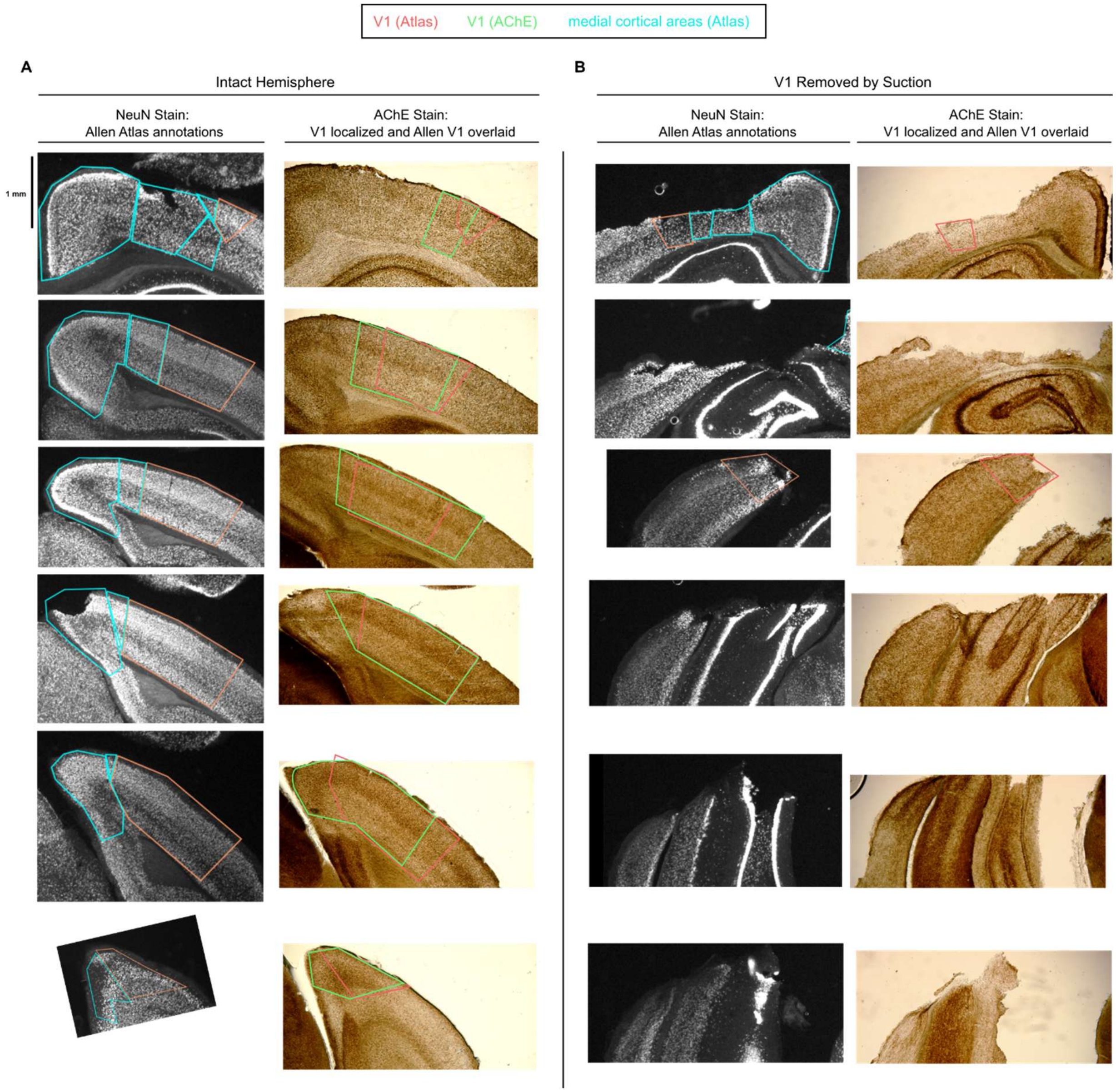

Figure S2. Localizing V1 using the Allen Mouse Brain Atlas corresponds well with localization using acetylcholinesterase (AChE) stain. Related to Figure 1.

(A-B) Serial coronal sections of mouse brain were stained for NeuN (left column) or AChE (right column). V1 was located in anti-NeuN slices by semi-automatically registering the serial slices to the Allen Brain Atlas (Methods) and is denoted with an orange outline. V1 was located in AChE-stained slices by the darkening induced in layer 4 of cortex and is denoted with a green outline. The anti-NeuN slice and AChE slice in the same row are sequential slices in the series. Each slice is 40 um thick and separated from the next slice in the next row by 80-400 um. Most anterior section is on top.

(A) Serial coronal sections from an intact brain. The right column shows the overlay between the orange and green outlines, yielding a good correspondence between the Atlas and AChE demarcation of V1.

(B) Serial coronal sections from a left V1-suctioned brain. The AChE images were taken of the most dorsal portion of cortex that remained. No AChE darkening is apparent in the remaining cortex, indicating complete removal of V1, as confirmed in the Atlas alignment showing the absence of V1 annotations on all sections except the first and third section. The AChE darkening visible in cortex in the fifth panel does not correspond to V1 but is likely primary auditory cortex. We quantified this brain as having 96% removal of V1 using the Atlas annotations.

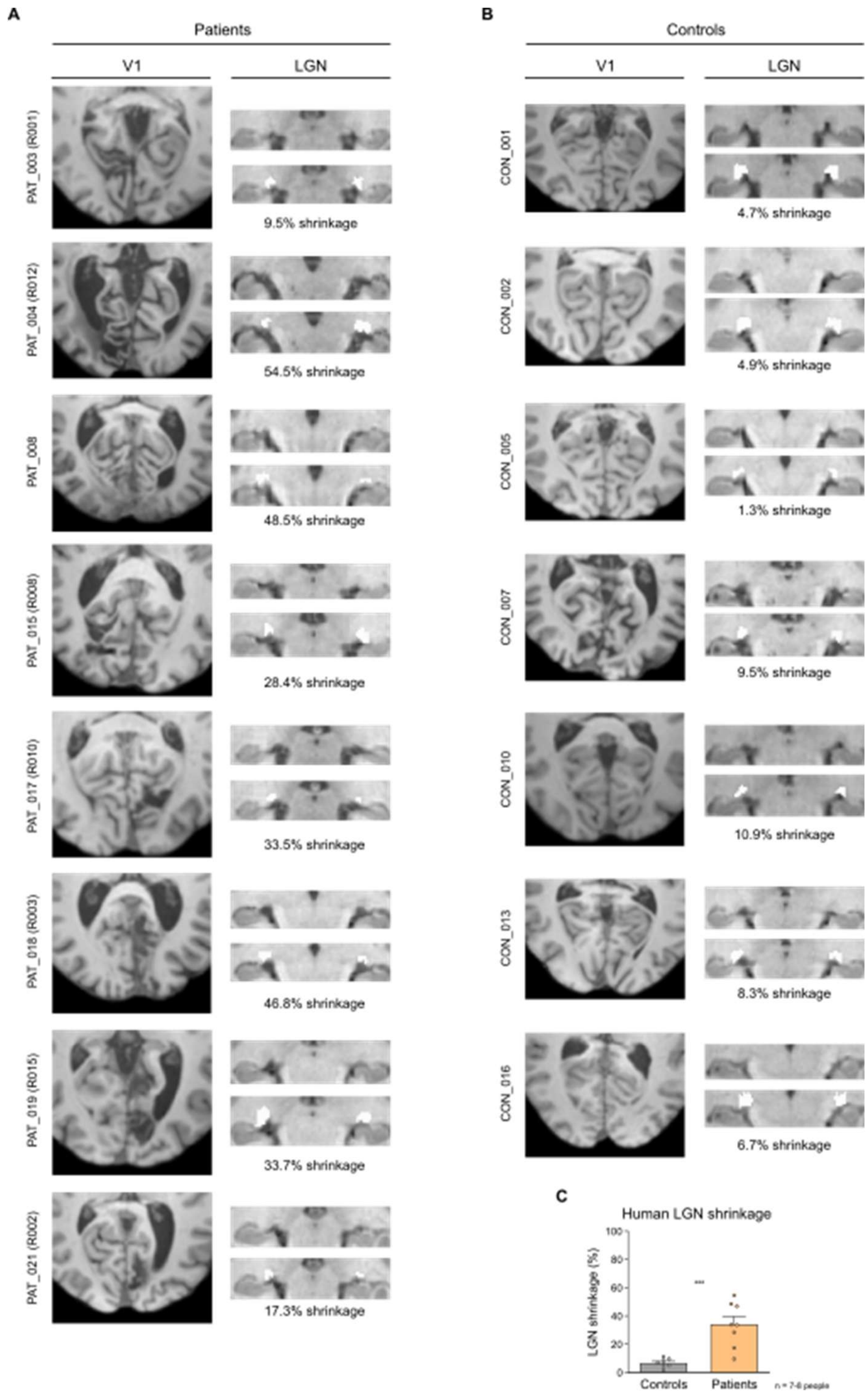

Figure S3. Human LGN degenerates by 34% after ipsilateral V1 damage. Related to Figure 1. (A-B) The left column shows a magnetic resonance (MR) image containing a horizontal section that includes right and left V1 for each patient (A) or control (B). The right column shows two identical coronal sections containing the right and left LGN for each person, with LGN annotated in white in the lower panel. For patients (A), the number below each annotated section is how much smaller the LGN ipsilateral to the lesioned V1 is relative to the contralateral LGN, as measured for the entire volume of each LGN. For controls (B), the number below each annotated section is how much smaller the smaller LGN is relative to the larger LGN, as measured for the entire volume of each LGN.

(C) Patients exhibit 34% shrinkage in LGN volume on average, whereas controls show a deviation between the left and right LGNs of 6.6% volume.

Each dot is one human. Error bars indicate SEM. *** $p < 0.001$, $n = 7$-8 humans.

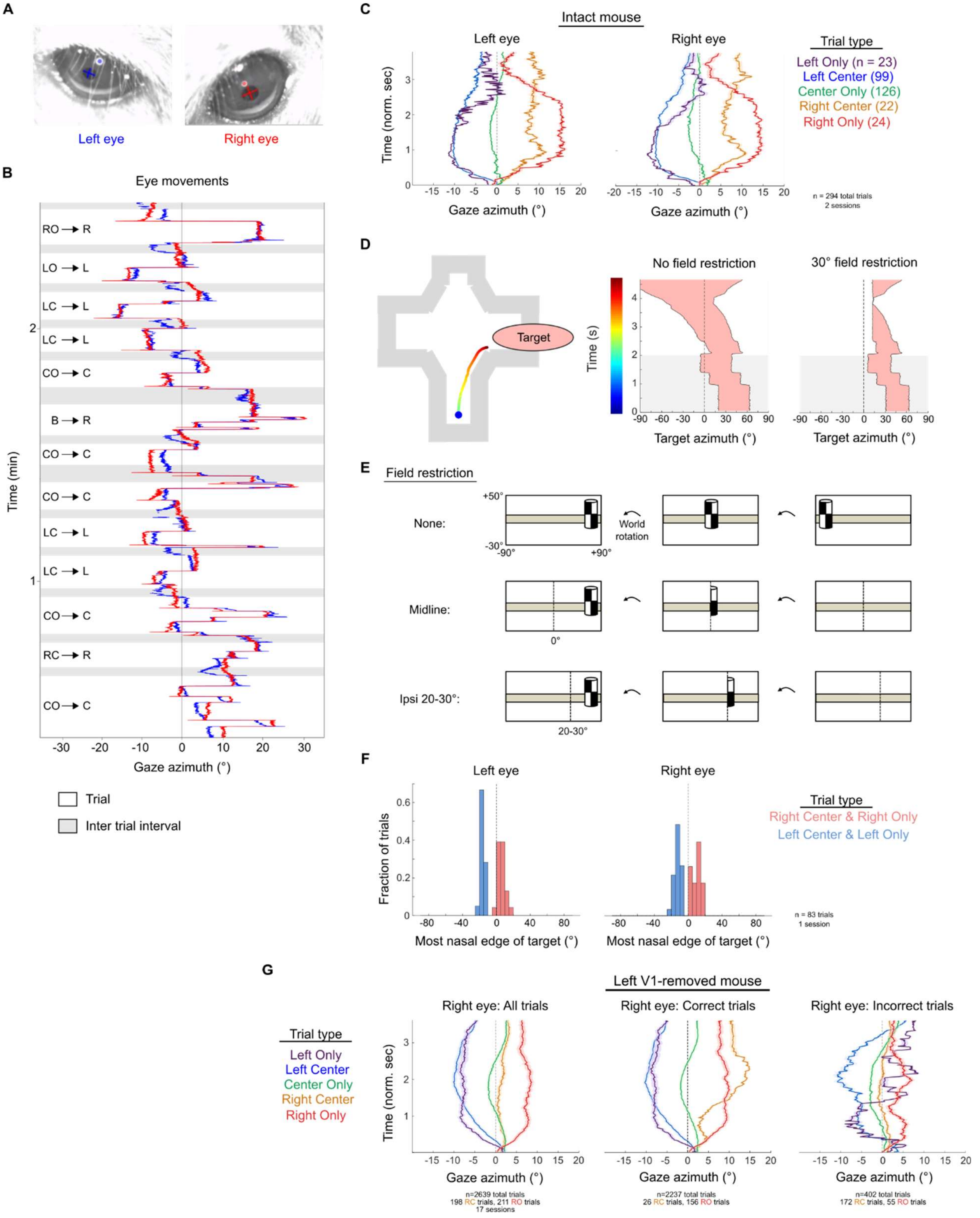

Figure S4. Mice move their eyes in the direction of the rewarded target after training. Related to Figure 1.

(A) Images of a mouse's eyes showing automatic annotations. Custom software automatically annotates the pupil (labeled with a '+') and the corneal reflection (labeled with a colored dot).

(B) Eyes move horizontally during trials in the direction of the rewarded target. Eye movements over approximately two minutes for one mouse. Left eye movements shown as blue traces, right eye movements shown as red traces. The initials preceding the arrow for each row indicates the trial type, with the initials after the arrow indicating the mouse's action on that trial; LC = left-center; LO = left-only, RC = right-center, RO = right-only, CO = center-only; L = left action, R = right action, C = center action.
(C) Eye movements track the rewarded target. When the target is on the left, the average eye movement is toward the left; when the target is straight ahead, on average there is very little eye movement away from the center; and when the target is on the right, the average eye movement is toward the right. Representative eye movements from an intact well-trained mouse, grouped and averaged according to the trial type for both eyes from two behavioral sessions. n = 294 trials.
(D) Target location in a mouse's field-of-view on one trial. Left, overhead view of the trajectory of one mouse on one right-only trial. The blue dot indicates the mouse's starting position, with varying colors of the running trajectory indicating the relative time the mouse is at that position. Middle, location of the target in the mouse's field-of-view during the trial. The grey region indicates the initial period of each trial in which movements on the ball are not translated into movements in the virtual world. The target starts in the right hemifield. Sharp changes in target location are due to eye saccades. As the mouse approaches the target, it fills more of the mouse's visual field and also expands into the left hemifield. Right, the same trace as before but now with field restriction imposed. Neither eye movements nor target expansion on approach result in the target entering the left hemifield.
(E) Effect of field restriction. Top: Mice are initially trained without any field restriction. Middle: Midline field restriction would prevent the target from being viewable in the opposite hemifield if the mouse's gaze was fixed, but because the gaze moves, midline field restriction is insufficient to prevent the target from being viewable in the opposite hemifield. Bottom: To account for gaze movement, which we observe to be between 20-30°, before surgery mice are trained with field restriction that enters 20-30° into the hemifield on the same side as the lateral target. They are then tested with the same field restriction following surgery.
(F) Distribution of the nasal edge of the lateral target across many trials following field restriction. For each eye, a histogram of the location of the most nasal edge of the left target on left target trials (blue) and the right target on right target trials (pink). If the target crosses the midline, as shown for one right target trial for the left eye, the mouse might be able to use their good visual field to demonstrate blindsight-like behavior. Therefore, to advance to surgery, at least 80% of trials must not have the lateral target crossing the gaze midline. n = 83 trials, 1 session.
(G) Following removal of left V1, when the mouse acts blind on right-center trials (right panel), the eyes move much less towards the right than prior to surgery (C). On the few right-center trials in which the mouse runs correctly to the right (middle panel), the average eye trajectory moves to the right, similar to before V1 removal (C).

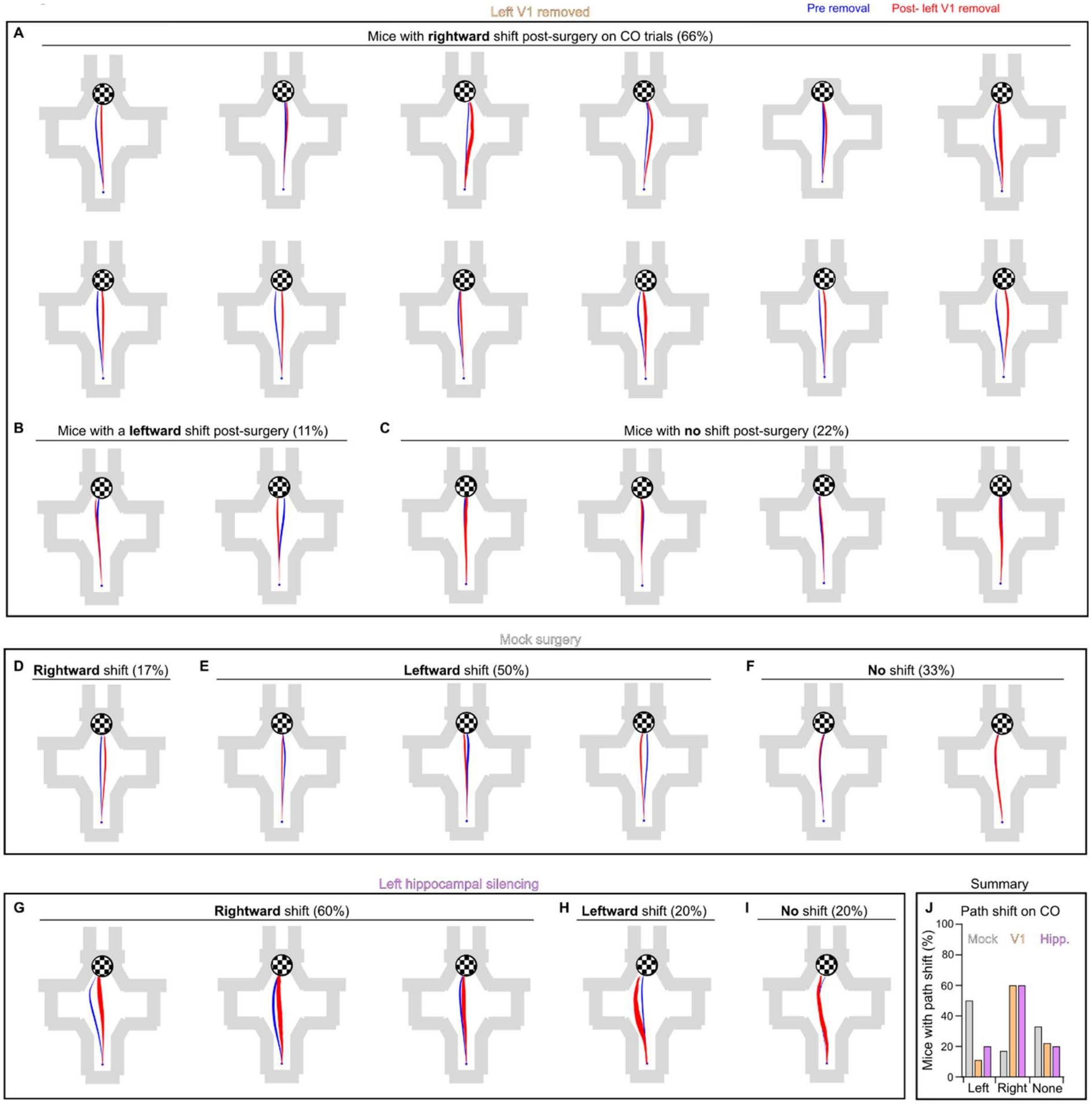

Figure S5. Removal of left V1 and silencing of left hippocampus causes a rightward shift in running paths consistent with hemianopia. Related to Figure 2.

Average running trajectories for each mouse on center-only trials. Mice start at the small blue dot at the bottom of each maze and run to the central target at the top. Paths before surgery are in blue, after surgery are in red; path line thickness indicates the 95% confidence interval. Thick grey lines indicate the boundary walls of the maze.

(A) After left V1 removal, 12 mice had a rightward shift in their paths.
(B) After left V1 removal, 2 mice had a leftward shift in their paths.
(C) After left V1 removal, 4 mice had no shift in their paths.
(D) After mock surgery (craniotomy but no suction), 1 mouse had a rightward shift in its path.
(E) After mock surgery, 3 mice had a leftward shift in their paths.
(F) After mock surgery, 2 mice had no shift in their paths.
(G) After left hippocampal silencing with muscimol, 3 mice had a rightward shift in their paths.
(H) After left hippocampal silencing, 1 mouse had a leftward shift in its path.
(I) After left hippocampal silencing, 1 mouse had no shift in its path.

(J) Summary of results. Difference between V1-removed and mock results: $p = 0.053$, Fisher exact test. Sample size of hippocampal silencing results is too small to get a statistically significant effect, but the trend matches V1 removal.

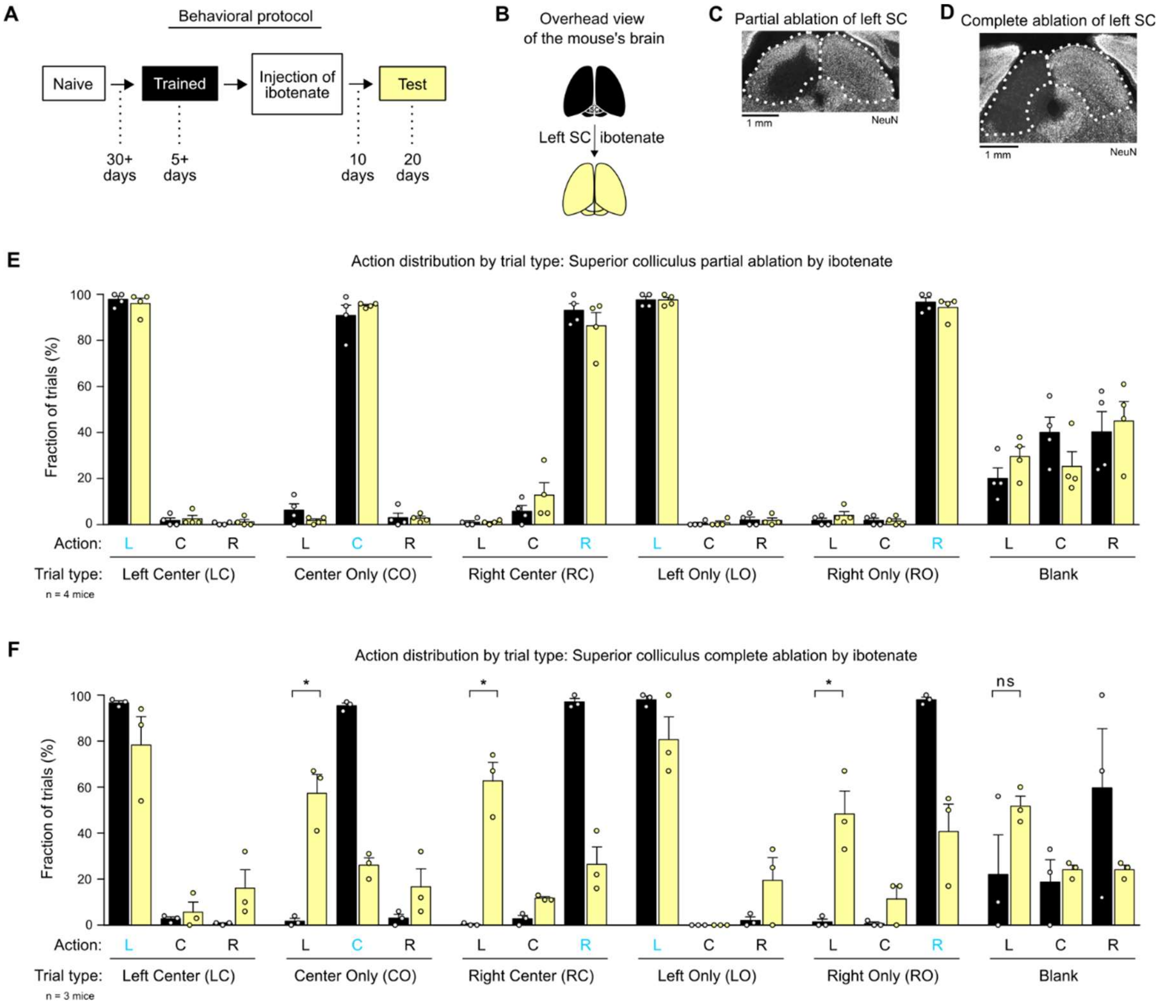

Figure S6. Pharmacological ablation of the left superior colliculus (SC) results in ipsiversive turning in virtual reality. Related to Figure 2.

(A) Behavioral training protocol. Trained mice are injected with ibotenate and tested on the task 10 days following surgery.
(B) Schematic of SC ablation with ibotenic acid.
(C) Coronal section showing partial ablation of left SC.
(D) Coronal section showing complete ablation of left SC.
(C-D) Both SCs are demarcated by a dotted line.
(E) Actions of mice on each trial type before (black) and after (yellow) partial ablation of left SC. Partial ablation of SC has little effect on behavior. n = 4 mice.
(F) Actions of mice on each trial type before (black) and after (yellow) complete ablation of SC. Complete ablation of SC causes a strong leftward bias on all non-left trial types, consistent with neglect. n = 3 mice.

Each dot is one mouse. Brain slices stained with anti-NeuN. Error bars indicate SEM. * $p < 0.5$, ns is $p \geq 0.05$, paired t-test comparing indicated groups.

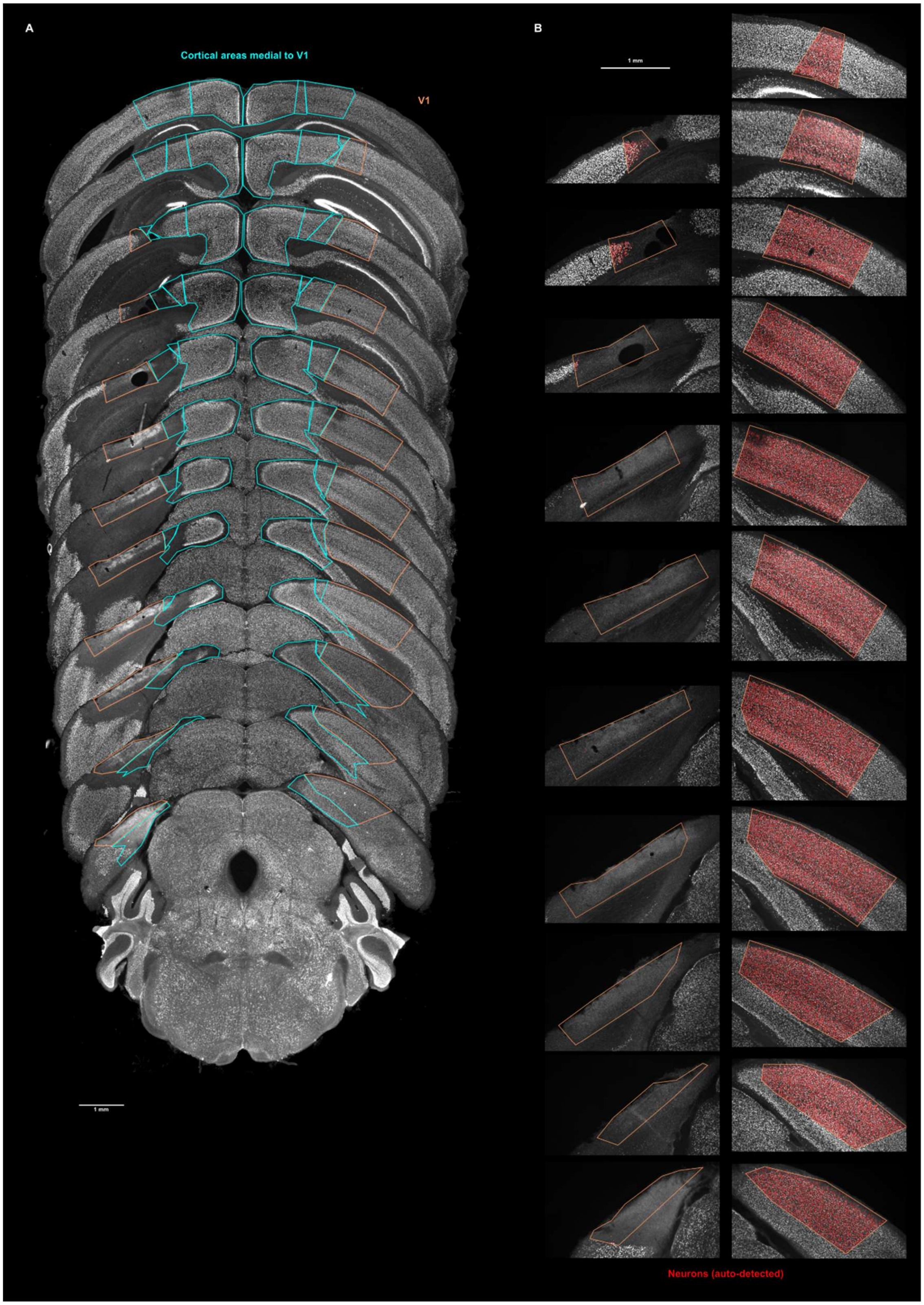

Figure S7. Serial coronal brain sections of a mouse with 99% neuronal loss in V1 after ibotenate injection. Related to Figure 5.

(A) Low-magnification serial sections of a mouse's brain. Each slice was 40 um thick, and the distance between slices was 80 um. To improve V1 localization, cortical areas medial to V1, including retrosplenial cortex (RSP), VisA, VisAM, and VisPM, were annotated (blue outlines) to ensure that the distance from the midline to V1 was the same as in the Allen Atlas.
(B) High-magnification serial sections of V1 (orange outline) on the injected side (left) and intact side (right). Neurons were automatically detected and outlined in red. 99% of neurons in left V1 were lost.

This mouse exhibited mostly normal vision with 70% RC adjusted accuracy. The most anterior section is on top. The white stain indicated neurons (anti-NeuN), except the diffuse white glow in left V1, which we believe to be an unexpected byproduct of ibotenate injection.
